# Emotion and motion: Toward emotion recognition based on standing and walking

**DOI:** 10.1371/journal.pone.0290564

**Published:** 2023-09-13

**Authors:** Hila Riemer, Joel V. Joseph, Angela Y. Lee, Raziel Riemer

**Affiliations:** 1 Guilford Glazer Faculty of Business and Management, Ben-Gurion University of the Negev, Be’er-Sheva, Israel; 2 Department of Industrial Engineering and Management, Ben-Gurion University of the Negev, Be’er-Sheva, Israel; 3 Kellogg School of Management, Northwestern University, Evanston, Illinois, United States of America; Ningbo University, CHINA

## Abstract

Emotion recognition is key to interpersonal communication and to human–machine interaction. Body expression may contribute to emotion recognition, but most past studies focused on a few motions, limiting accurate recognition. Moreover, emotions in most previous research were acted out, resulting in non–natural motion, which is unapplicable in reality. We present an approach for emotion recognition based on body motion in naturalistic settings, examining authentic emotions, natural movement, and a broad collection of motion parameters. A lab experiment using 24 participants manipulated participants’ emotions using pretested movies into five conditions: happiness, relaxation, fear, sadness, and emotionally–neutral. Emotion was manipulated within subjects, with fillers in between and a counterbalanced order. A motion capture system measured posture and motion during standing and walking; a force plate measured center of pressure location. Traditional statistics revealed nonsignificant effects of emotions on most motion parameters; only 7 of 229 parameters demonstrate significant effects. Most significant effects are in parameters representing postural control during standing, which is consistent with past studies. Yet, the few significant effects suggest that it is impossible to recognize emotions based on a single motion parameter. We therefore developed machine learning models to classify emotions using a collection of parameters, and examined six models: k-nearest neighbors, decision tree, logistic regression, and the support vector machine with radial base function and linear and polynomial functions. The decision tree using 25 parameters provided the highest average accuracy (45.8%), more than twice the random guess for five conditions, which advances past studies demonstrating comparable accuracies, due to our naturalistic setting. This research suggests that machine learning models are valuable for emotion recognition in reality and lays the foundation for further progress in emotion recognition models, informing the development of recognition devices (e.g., depth camera), to be used in home-setting human–machine interactions.

## 1. Introduction

### 1.1. Emotion and body motion

Emotions are a vital part of communication. Emotion recognition is key to understanding people’s motivations and behaviors and is therefore an essential part of human–machine interaction [1]. Emotions can be recognized through the ‘translation’ of non-expressive verbalization or voice modulation [2, 3], facial expression [4–6], or body language [7, 8].

Embodiment theories [9] focus on the reciprocal relationships between emotions and body motion. Indeed, early studies from the mid-1960s [10] provided evidence for the contribution of body cues to emotion recognition. More recent studies have suggested that body expressions can add accuracy to emotion recognition and may be useful when facial expressions and voice data are not accessible [8, 11]; body expressions are also prioritized over facial expressions when the body and face are incongruent [12, 13].

To inform the development of emotion recognition devices in human–robot interactions, we present an approach for emotion recognition based on body motion in naturalistic settings. The relationships between emotions and body motions have been studied from various perspectives and approaches mainly using traditional statistics or machine learning algorithms. The current study combines both approaches by conducting a controlled lab experiment mimicking home settings where human–robot interactions might take place.

### 1.2. Experimental studies on emotion and motion

Experimental studies on emotion recognition typically use various operationalizations of emotion followed by measurements of body motion. The operationalization of emotions in past studies have relied on emotion-inducing stimuli (e.g., pictures [14]) or environment to manipulate emotional state (e.g., an elevating standing surface inducing anxiety [15]); often, operationalization has involved asking participants to act out moving under certain emotional states (e.g., [16]). Measurements of body motion have relied either on observers who coded various ‘qualitative’ motion factors [17], on video cameras [18], or on motion capture systems [19]. The link between the emotional state and body motion in such studies was analyzed using traditional statistics, examining associations between emotion and body motion parameters, including: postural control during standing [20], walking parameters (e.g., walking speed, stride length, arm swing, head movement, heavy footed, posture, (e.g., [16]), and gait initiation (e.g., [21]), as discussed below.

Studies on emotional effects on postural control during standing have focused mainly on states of anxiety and fear of falling (see reviews by Adkin and Carpenter [22] and by Lelard, Stins, and Mouras [23]). This line of research generally shows that these states reduce sway, perhaps due to changes in balance control strategy. Studies on postural control in response to visual stimuli have demonstrated mixed effects of unpleasant stimuli on postural control: some reported freezing or stiffness ([14, 24–26], and others reported withdrawal [27, 28]. Horslen and Carpenter [20] disentangled the effects of the arousal and valence dimensions of emotions on postural control and showed that it is the arousal dimension, but not the valence dimension, that leads to effects on body postural control. Interestingly, however, Mouras, Lelard, Ahmaidi, and Krystkowiak [29] demonstrated enhanced postural control under conditions of exposure to sexual stimuli as compared to humorous stimuli; this indicates that it is not only heightened arousal that influences postural control, but rather the specific emotion.

Research on the link between emotions and walking parameters consistently showed that sadness and depression are associated with reduced walking speed, arm swing, and vertical head movement and with both larger upper body sway and collapsed posture [30]. Yet, with regard to step initiation the findings are mixed: some showed that pleasant stimuli facilitate step initiation [31–33] whereas others showed facilitation by unpleasant stimuli [34]. These inconsistent findings are partly due to differences in the timing of the emotional processing, such that the appearance of pleasant stimuli and disappearance of unpleasant stimuli would lead to faster gait initiation [35]. Furthermore, Bouman, Stins, and Beek [21] showed that it is the arousal dimension of the emotion, but not valence, that influences the kinematic of forward gait initiation.

Experimental studies of emotion *recognition* from body motion (i.e., beyond identifying emotion-motion links) have involved asking participants to act out emotions in their body motion, with observers then asked to recognize these states. Montepare et al. [16], for example, showed that walking gait parameters—arm swing, stride length, and walking speed—enhance the identification of sad, angry, happy, and proud emotional states, with lower accuracy for pride. Wallbott [18] asked participants to act joy, happiness, sadness, despair, fear, terror, anger, disgust, contempt, shame, guilt, pride, or boredom, and measured a broad collection of motion parameters. He showed that discriminant analysis enables emotion classification with an accuracy that is far larger than chance. Roether et al. [36] showed that walking speed, posture, and dynamic are critical for observers’ emotion recognition. Importantly, De Meijer [37] indicated that trunk movement, arm movement, upward/downward movement, forward/backward movement, as well as force and velocity in the sagittal direction are valuable to emotion recognition; Importantly, however, he suggested that it is not a single specific motion but rather a combination of motions that contributes to the recognition of emotion. Gross et al. [19] explored a wide variety of motion parameters during walking with a combination of approaches. First, they manipulated participants’ emotions (to joy, contentment, anger, sadness, or neutral) and instructed them to walk *as they express their emotions*. They then asked observers to identify emotional states and to provide a qualitative description of the effort-shaped movement characteristics. They also used a motion capture system to measure quantitative body posture and limb motion during walking. Interestingly, although participants were encouraged to express their emotions, the results revealed significant differences in only a few of the quantitative motion features. Moreover, more differences were shown in the qualitative analysis as compared to the quantitative analysis. This led the researchers to conclude that observer judgment is more sensitive to motion differences under emotional states than measurements obtained using technological devices. These insights justify examination using more complex models, as described next.

### 1.3. Using machine learning for emotion recognition from motion

As opposed to traditional statistics, machine learning methods are used for modeling, prediction, and detection, based on experience by mapping the input data to the output [38–40]. These methods are useful for complex phenomena involving many parameters—some might be inter-related—where no explicit rules are available [41]. Advanced versions of machine learning (e.g., convolutional neural network; CNN) enable complex modeling, which can generate relatively high prediction accuracy.

Studies utilizing machine learning methods to identify people’s emotional states have used algorithms such as support vector machines (SVM), random forests, and neural networks [42–47]. These studies relied on parameters of specific motions (e.g., gait [2, 3], [43, 45], dancing [48, 49], knocking [50–52], performing tennis movements in a Wii video game [51], or body posture (3). As an input to the algorithms, some researchers have used traditional biomechanical parameters, such as stride length, cadence, speed, and joint angles [2, 43]; others have used less intuitive parameters, such as joint location, joint angle, derivatives of joint parameters, relative distances between markers, and Fourier transformation. The number of parameters used as input to the classifiers has ranged from dozens [2] to hundreds [46]. Most algorithms are used as black box methods, where the relation between given motion parameters and the emotion is not known (e.g., SVM [2], [49, 50], naïve Bayes [2], and the recurrent neural network algorithm [51]).

Studies that have employed such algorithms to identify emotional states based on motion parameters reached accuracy rates of 50%–80% (see Table 1). Yet, these studies were limited in the ways in which the ‘ground truth’ (i.e., the actual emotional state associated with the various movements) was determined. Two issues contribute to the artificial nature of the studies: First, most studies used participants who acted out the emotional states through their body motion, which might lead to exaggerated movement [17, 37, 43, 45, 48, 52]. Second, many studies relied on observers, who rated the extent to which the body motion represented a specific emotion [2, 3, 42, 47]. This latter approach is limited, as the agreement between an observer rating and the actual expressed emotion has been shown to be only approximately 65% [2, 3]. Furthermore, observers used their own (naïve) perspective on how emotion should be expressed, which may have been less accurate than participants’ self-reporting of their emotions. These limitations make it difficult to extrapolate findings to more natural settings. To the best of our knowledge, only one machine learning study relied on a natural setting: Zhao et al. [46] measured participants’ anxiety and depression levels using validated questionnaires and captured participants’ walking using a Kinect camera. They then tested several algorithms, where Gaussian processes achieved the best overall accuracy of 43% predicting anxiety level (when the model was trained for females only, it achieved an accuracy of 61%, and when trained for males only it achieved an accuracy of 71%). Prediction accuracy levels for depression were 51% overall, and 45% and 64% when trained separately for females and males, respectively.

**Table 1:** Studies that used machine leaning for emotion classification based on body motion.

Authors [Reference number]	Number of participants	Emotion operationalization	Classified emotions	ML methods	Range Average accuracy
Camurri et al. 2003,2008, [49, 53]	5	Dancers **acting** emotions, and **observers** identifying the emotions	Anger, fear, grief, and joy	Decision tree	40%
Park et al. 2004 [48]	4	Dancers **acting** emotions	Happiness, surprise, anger, and sadness	Terrace distribution map for the loess plateau (TDMLP)	73%
Gong et al. 2010 [50]	30	Non-professional actors **acting** emotions	Neutral, happiness, anger, and sadness	RBF kernel SVM, with different feature manipulations	50–76%
Karg et al. 2010 [2]	13	Participants **acting** emotions	Anger, happiness, neutral, and sadness	Naive Bayes, Nearest neighbor, SVM	41–69%
Kleinsmith et al. 2011 [3]	11	Videotaping players during a Nintendo Wii game, and **observers** identifying the emotions	Concentration, defeat, frustration, and triumph	*Multilayer perceptron* (*MLP*)	52%
Savva and Bianchi-Berthouze 2011 [51]	9	Videotaping players during a Grand Slam Tennis Wii game and **observers** identifying the emotions	High intensity negative, happiness, concentration, low intensity	Recurrent Neural Network algorithm RNN	57%
Wang et al. 2015 [50]	11	Professional actors **acting** emotions and **observers** identifying the emotions	Fear, happiness, anger, and sadness	Random Forest with different groups of features	57–77%
Daoudi et al. 2017 [43]	8	Professional actors **acting** emotions	Anger, fear, joy, neutral, and sadness	Nearest-neighbor and Riemannian manifold of SPD matrices	51%,71%
Randhavane et al. 2019 [42]	5 data sets of 11, unknown number, 24, or 30 motions of real people, and 41 with synthetic movement	**Observers** identifying the emotions in movie segments from various data sets	Happiness, anger, sadness, and neutral	Long Short-Term Memory (LSTM) Networks + SVM or random forest	70–80%
Zhao et al. 2019 [46]	179	Participants completed scales measuring depression and anxiety disorder	Anxiety and depression	Epsilon-Support Vector Regression, Gaussian Processes,	51–74%

Altogether, the accumulated research suggests a possibility of recognizing emotional state based on body motion. Yet, most studies so far have focused on a limited number of motion parameters, constraining the accuracy of emotion recognition, or were conducted in an artificial setting relying on acted movement and/or observes. Our study was designed to address these limitations.

### 1.4. The current study

To address the gaps in past studies, the current research explores the possibility of accurately determining one’s authentic (i.e., non-acted) emotional state based on natural human movement. In light of the findings of past studies, we expect that the effects will be rather minor, which may not enable accurate emotion recognition based on single parameters. Therefore, we explore a broad collection of body motion and posture parameters. We examine emotion and motion without overt expression, exploring a wide variety of motion parameters during standing (posture and balance) and walking (gait and posture parameters), which may assist in identifying which ones contribute to accurate emotion recognition.

Both conventional statistics and machine learning were used for the analysis. This will assist in uncovering not only effects on specific motion (which are not expected to enable identification of emotion [19, 37]), but to examine the extent to which a collection of motion parameters enables emotion classification. In addition, since past research has indicated gender differences in the experience and expression of emotions [54, 55], we explore the role of gender in the effects of emotion on motion. To these ends, we conducted a lab experiment manipulating emotions by using pretested movie segments and measuring body motion by using a force plate and motion capture system. The experimental setting mimicked the home setting, where human–robot interactions usually take place. Results from this study may inform developers of emotion recognition devices, which can be useful in the development of social robots and improve human–machine interaction.

## 2. Method

### 2.1. Experimental design and sample

A lab experiment used a within-subjects approach, where emotional state was manipulated into five conditions (four emotional conditions and a neutral condition). A motion capture system and a force plate measured body motion. Participants were 24 students (10 male; *M*_age_ = 25.06 years) recruited through our student subject pool; participation was restricted to healthy people with no injuries or movement impairments. Sensitivity analysis for this sample size was conducted using G*power 3.1.9.7. This analysis was performed considering ANOVA for within-subjects design—with five conditions (neutral, and four emotional conditions), α of 0.05, and power of 0.8. The sensitivity analyses revealed that our sample size can detect effects sizes f larger than .23, which is considered a medium effect size. Thus, our sample can detect effects of medium or large size. Our Institutional Review Board approved the protocol (#HR20122012), and the participants read and provided written informed consent.

### 2.2. Emotion manipulation

The emotional states varied in terms of valence and arousal dimensions, namely happiness (positive, high arousal), relaxation (positive, low arousal), fear (negative, high arousal), and sadness (negative, low arousal) [56], with filler tasks in between. We also added an emotionally neutral condition. In line with past research [57], four movies were used to manipulate the participants’ emotional states. The movie selection was based on a manipulation check conducted on a pretest using other participants recruited from the same student subject pool used for the main experiment. The pretest used a between-subjects design, and the four selected movies were rated by 80 participants (20 participants in each condition). Pretest participants watched movie segments intended to elicit different emotions. They reported their emotional responses to each segment by using the Mehrabian and Russell scale [58] consisting of six bipolar items measuring the valence dimension (happy–unhappy, pleased–annoyed, satisfied–unsatisfied, contented–melancholic, hopeful–despairing, relaxed–bored) and six bipolar items measuring the arousal dimension (stimulated–relaxed, excited–calm, frenzied–sluggish, jittery–dull, wide awake–sleepy, aroused–unaroused); each item was rated on a 9-point scale (without number notations on the scale). We identified four movie segments (duration: 120–193 s) that elicited the intended emotions (Table 2).

**Table 2:** Emotion manipulation movie segments. Valence and arousal scores are on a 1–9 scale. Values in parentheses are standard deviations.

	Negative valence	Positive valence
High arousal	State: **Fear**	State: **Happiness**
	Name of the movie: Silence of the Lambs	Name of the movie: The Untouchables
	Description of scene: A female officer with a gun looks for a suspect in his dark house	Description of scene: Characters are singing, dancing, and enjoying themselves
	**Valence score = 4.04** (1.54)	**Valence score = 7.58** (1.35)
	**Arousal score = 6.86** (1.30)	**Arousal score = 6.42** (.92)
Low arousal	State: **Sadness**	State: **Relaxation**
	Name of the movie: The Champ	Name of the movie: Nature clip
	Description of scene: A child is crying upon seeing his boxing hero dead after a match	Description of scene: A short clip with nature, animals, and calm music
	**Valence score = 3.08** (.93)	**Valence score = 6.82** (1.40)
	**Arousal score = 4.89** (1.44)	**Arousal score = 3.29** (1.39)

The manipulation was confirmed in an additional manipulation check in the main experiment. For the additional check, at the end of the main experiment, participants were provided with a cue for the movies they had watched earlier in the session; they were asked to indicate the extent to which they felt each of the four target emotions after watching each segment. This measure used a 7-point scale and included questions similar to those in the PANAS scale of affective state [59] but was adapted to include only the four target emotions (sadness, happiness, relaxation, and fear).

The order of the four emotional conditions in the main experiment was randomized through OpenSesame [60]. The neutral state was included as the first condition for all participants. The rationale for why this neutral state was first for all participants comes from the assumption (based on our experience with manipulating emotions in lab experiments) that transitions between emotional states are likely to be successful with filler tasks along with the emotion manipulation; by contrast, the transition to a neutral state may not be successful using filler tasks alone, as these may not be sufficient to bring participants back to their neutral condition.

### 2.3. Procedure

To prevent hypothesis-guessing bias and conscious effort to exhibit emotions, the experiment’s purpose was disguised until the end of the session. Participants were told that the study was intended to examine the relationship between physical and cognitive factors.

**Fig 1:**
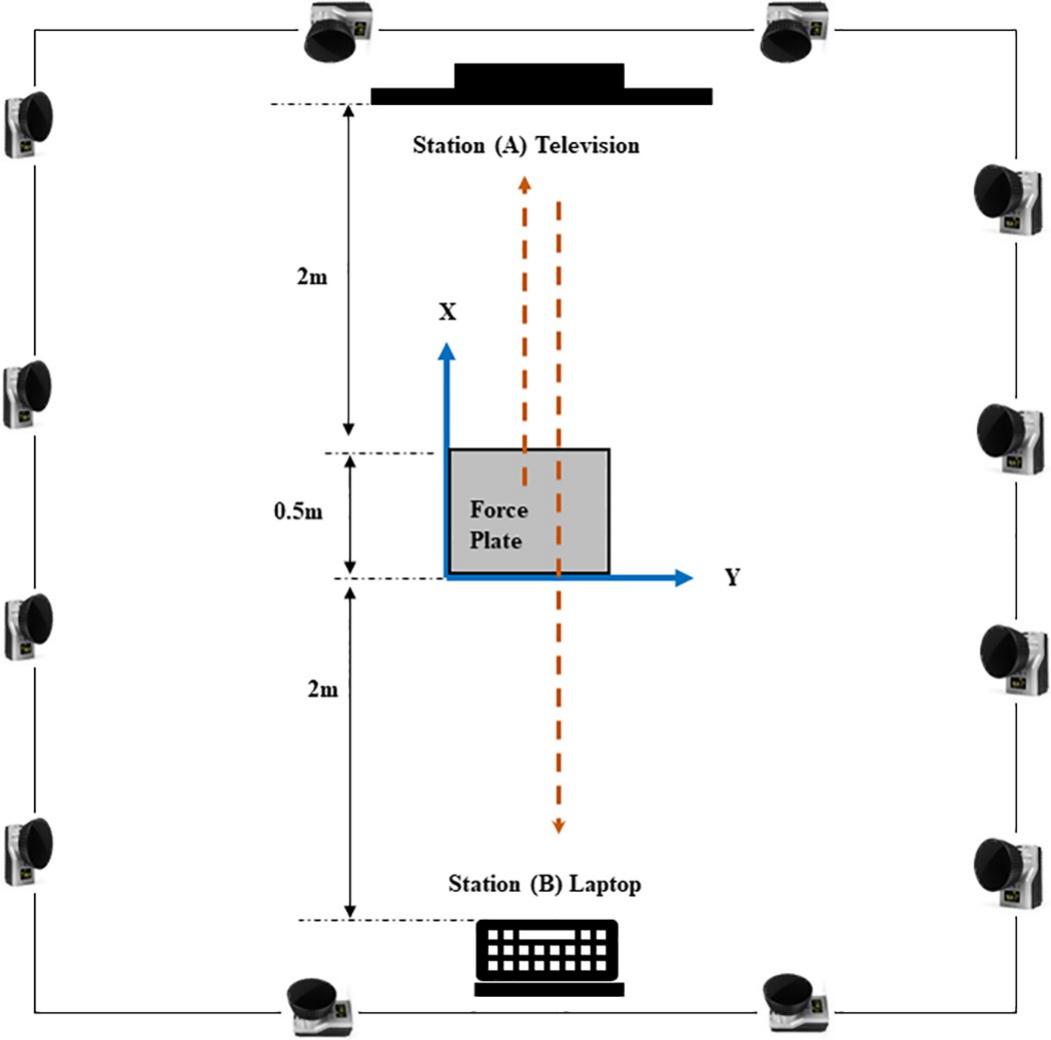
Lab setup. The figure is an illustration only, and the measures are not in scale.

The experimental setup (Fig 1) consisted of a starting station (the force plate on which the participant stood while watching the movies) and two computer stations (A and B). Stations A and B were 4.5 m apart, and gait movement was recorded while participants walked between these points. The distance between the stations mimics home settings where human–robot interactions typically take place. Station A included a 55-inch TV screen placed 2 m from the force plate; this was used to play the instructions and the movies. At station B, participants sat to perform filler tasks between emotional conditions. The filler tasks included reading emotionally neutral texts, answering questions about the reading texts, as well as completing behavioral and psychological tendency scales (e.g., personality scale, cultural orientation, which have not been used for the current analysis).

Participants first performed four practice sessions. In each practice session, they watched a blank screen for 10 s while standing on the force plate; they walked to station A and then to station B. During the actual experiment, participants read the instructions on the TV screen and moved to stand on the force plate; the movie segment began 5 s later. When the segment ended, participants walked to station A, pressed a button, and walked to station B, where they sat and performed filler tasks. Participants then moved back to the starting position for the next condition. The final task involved the additional emotion manipulation check (described above) to determine whether the intended emotion had been successfully evoked.

### 2.4. Motion measures

**Fig 2:**
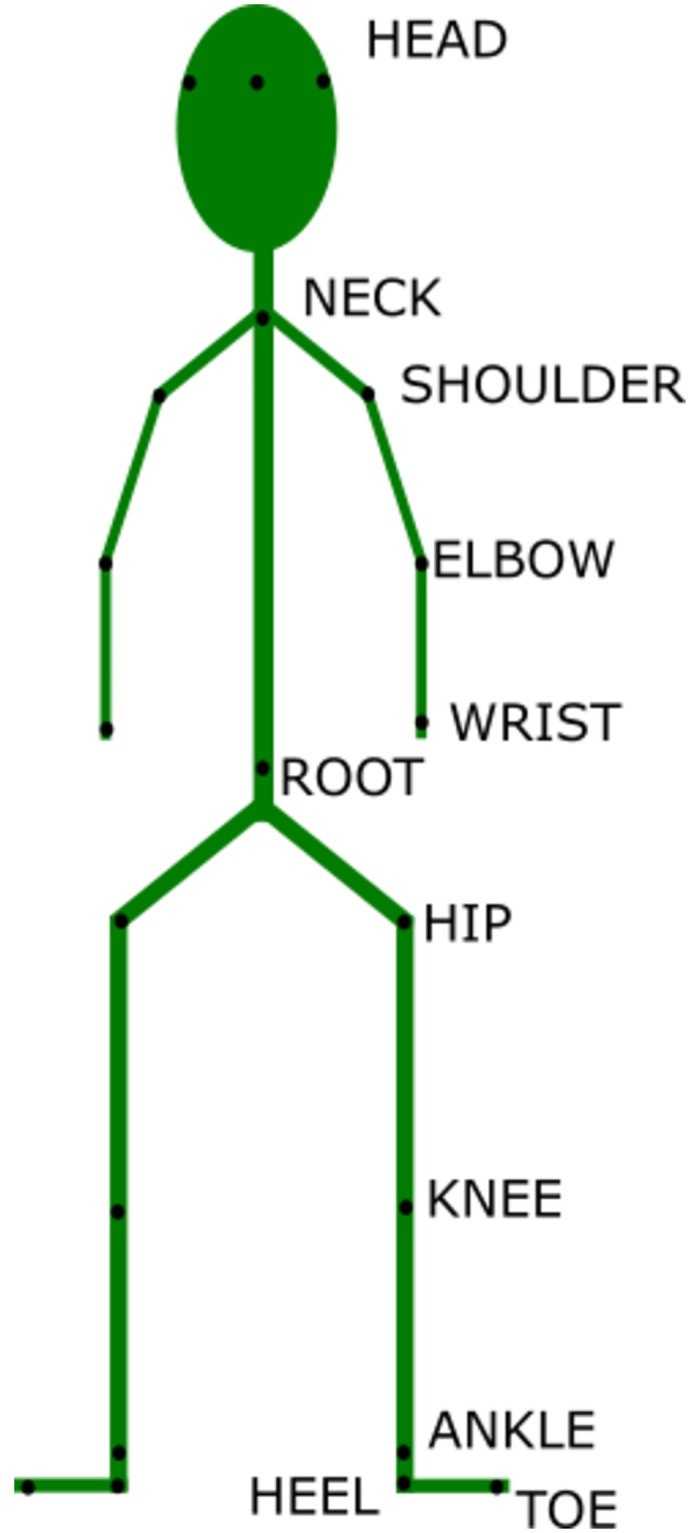
Locations of markers for measuring body motion using a motion capture system.

Body motion was recorded while the participants stood watching the movies and while they walked from the force plate to station A and then to B after watching each movie. We used a motion capture system with 14 cameras (Oqus 300 and Oqus 500, Qualisys, Goteborg, Sweden). Twenty-two reflective markers were placed on each participant’s body (Fig 2). Ground reaction forces and center of pressure (COP) were recorded while participants stood watching the movies by using the force plate (OR6-7-2000, AMTI, MA, USA). Marker data were captured at a rate of 120 Hz; force plate data were captured at 1000 Hz and later synchronized with the marker data [61, 62].

#### 2.4.1 Motion parameters

The recordings of each session were segmented into standing (30 s from the beginning of the movie to 5s before the participant moved off the force plate), gait initiation (the first two steps taken off the force plate toward station A), and walking (all full gait cycles after turning away from station A and walking to station B).

In the standing phase, the force plate obtained measurements of each participant’s COP, which were then used to calculate measures indicative of balance, such as the standard deviation (SD) in the anterior–posterior and mediolateral directions as well as the velocity and its SD. The motion capture data during the standing phase were used to calculate each participant’s base of support and step width as well as postural parameters such as the mean and SD of the upper body angles (for definitions, see S1 Appendix). In the gait initiation phase, we calculated the length, duration, speed, height, and width of each participant’s first two steps. In the walking phase, we calculated measures similar to those for gait initialization for the left and right gait cycles. We also calculated the mean and SD of various upper body angles.

Relying on past studies [36, 37, 45], we calculated 229 measures classified into four categories: balance (i.e., movements of the COP), standing posture (i.e., upper body angles), gait initiation, and walking (S1 Appendix). For each phase we corrected for potential biases, as follows.

*Balance*. People may shift their weight between legs while standing. To remove the effect of this shifting on calculations related to body sway, we used a combination of clustering methods (mean shift and density-based spatial clustering of applications with noise, hereafter DBSCAN [63, 64]. Mean shift was used to identify COP locus during standing, but since this also includes transitions, DBSCAN was employed on each cluster to remove the transition phases. We then calculated the weighted average and SD of the parameters relative to the number of data points in each cluster (COP transition to a new locus).

*Standing posture*. People may move their hands when standing still (e.g., touch their face). To remove such transitions from the analysis, we employed a clustering method similar to that used for the balance phase. The weighted average and SD of the parameters were calculated relative to the number of data points in each cluster.

*Gait initialization*. The first two steps were identified using a combination of gait event detection and force plate data. Parameters, such as step length and step time, were normalized based on the participant’s leg length [65], determined for each step by using motion capture data.

*Walking*. In accordance with past research, the walking parameters were calculated for each leg separately and normalized [65]. These parameters were taken as aggregates of the full steps performed during walking (2–3 per participant). The gait events (heel strike and toe-off) were identified using an established modification of the coordinate based treadmill algorithm (CBTA) method [66–70], by which the maximum distance between the hip (great trochanter) and the heel markers during the gait cycle is defined as a heel strike, while the minimum distance between the hip marker and toe marker defines the toe-off event. Walking posture parameters were also calculated and aggregated.

It should be noted that the 229 parameters include different modifications of similar core motion parameters. For example, we created two versions of COP motion parameter: one while taking into account that some participants may shift their weight between legs, and a second ignoring such shifts; as another example, we included both normalized and non-normalized step length.

#### 2.4.2. Data processing and analysis

The recording of each condition session was considered a trial, with five trials per participant: one neutral trial and one for each of the four emotions. Each recording was manually trimmed from its start to 0.5 s before the participant reached station B. In all, three trials for the relaxed condition and one for the fear condition were removed from the analysis due to recording errors.

**Fig 3:**
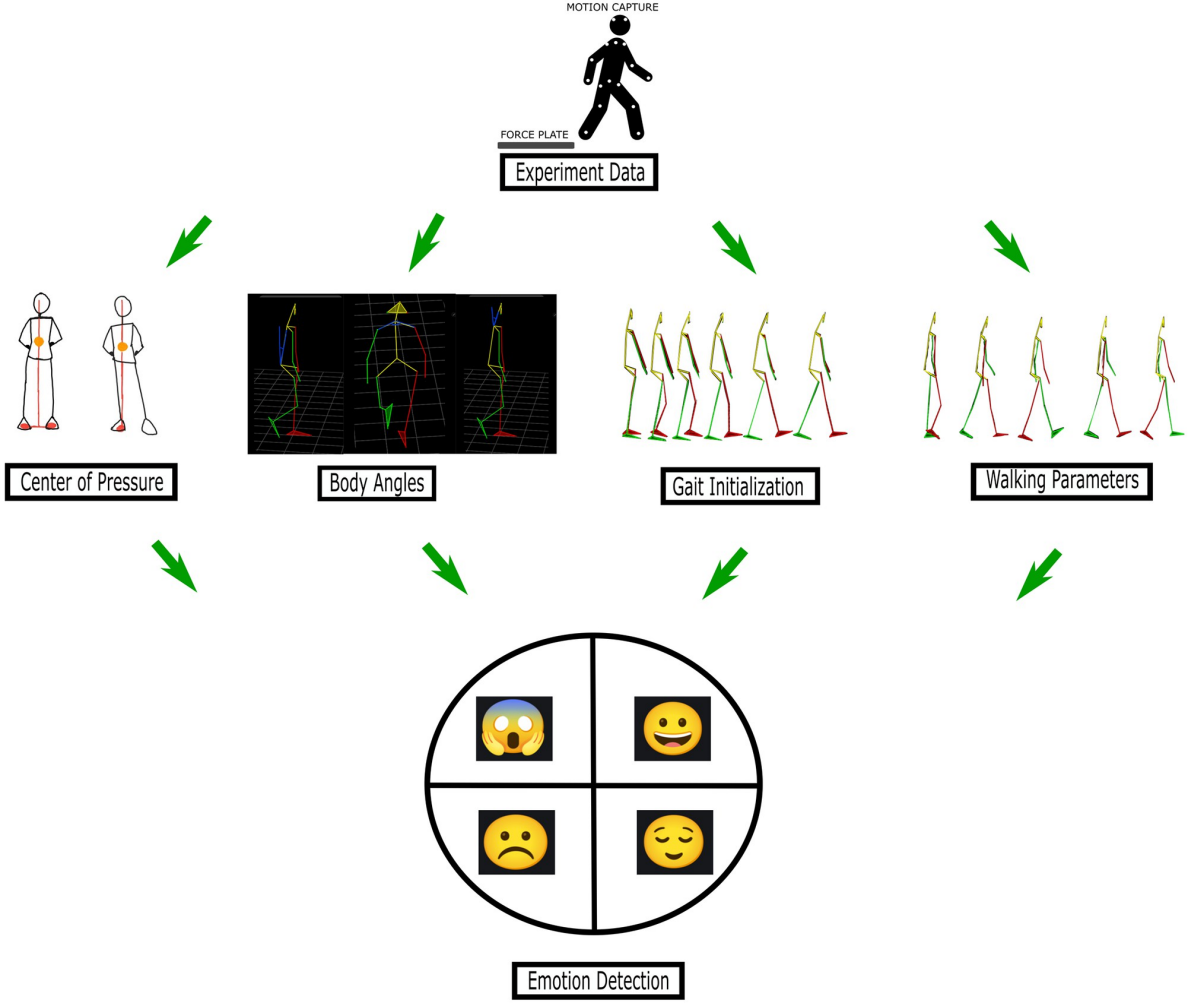
Data processing pipeline.

Motion data were cleaned and gap-filled using skeleton constraints and model averaging [71]. The data were filtered using a second-order Butterworth filter with a cut-off frequency of 10 Hz and 20 Hz for motion and force, respectively [72–74]. The filtered data were processed using a custom MATLAB program that identified the phases (Fig 3).

### 2.5. Statistical testing

Since many parameters were not normally distributed and given the small sample size, we used nonparametric permutation testing. This approach does not rely on distributional assumptions and utilizes resampling methods without replacement to generate the null hypothesis sampling distribution [75] The analysis [76] was performed using the open-source software Permuco (version 1.1.1; R Project). The test, in our case, was a randomized repeated measures ANOVA with 10,000 runs and post hoc pairwise comparisons between different emotions using a randomized t-test with 1000 runs with false discovery rate adjustments [77–80]. To explore the role of gender, we also used two-way ANOVA with a similar approach. P-values smaller than 0.05 were considered statistically significant.

### 2.6. Machine learning

To examine how various machine learning models might determine the participants’ emotional states from motion measures, the data were split into two parts: one for training the machine learning algorithm (80%) and another for testing the model’s prediction (20%). Both the training and testing data were sampled from the same pool, while maintaining the ratio of sampled data from each emotion condition similar to the ratio in the raw data and across the training and testing (i.e., stratified sampling).

We tested the following models: k-nearest neighbors (kNN) [81, 82], decision tree [83], logistic regression [84], and the support vector machine (SVM) [85] with radial base function (RBF) and linear and polynomial functions. For more on machine learning models and their application in biomechanics, see [39]. Since this is a multi-class classification task, the models were evaluated in terms of their accuracy, precision, recall, and F1 score. Accuracy was defined as the proportion of correctly classified points out of the total (random classification in our case of five emotional states would be 20%); precision was the proportion of predicted positives that were actually positive; recall was the proportion of actual positives that were classified correctly; and the F1 score was the harmonic mean of precision and recall.

## 3. Results

### 3.1. Manipulation check

For each movie segment, we used the data of the manipulation check conducted in the main experiment to compare the score for the intended emotion to those for the other emotions. We also compared the score for the various emotions across movies. The scores are presented in Table 3. The statistical test revealed significant differences in all cases (*p* < 0.05). Thus, consistent with the manipulation check conducted in the pretest, this additional manipulation check confirmed that the movies indeed evoked the intended emotions.

**Table 3:** Results for the emotion manipulation check conducted during the main experiment. Emotion ratings are on a 1–7 scale where 1 = not at all and 7 = very much.

	Fear rating	Happy rating	Relaxed rating	Sad rating
Fearful movie	**5.34**	1.52	1.30	2.56
Happy movie	1.32	**5.60**	3.32	1.76
Relaxing movie	1.20	5.45	**6.7**	1.45
Sad movie	3.91	1.16	1.54	**6.12**

### 3.2. Effect of emotion on specific motion parameters

Results of the statistical analyses performed for each motion parameter separately revealed that of the 229 parameters assessed, only 7 demonstrated significant effects. Six parameters showed significant main effects of emotion on motion, and an additional parameter exhibited a significant moderating role for gender in the effect of emotion on the motion. As presented in S2 Appendix, the following six measures—SD of the COP movement in the mediolateral (Y) axis during standing, SD of standing shoulder XY angle (sagittal plane), SD of standing shoulder XZ angle (frontal plane), SD of standing back angle (sagittal plane), SD of standing left wrist to hip distance, and SD of standing right wrist to hip distance—demonstrated significant differences only between the neutral condition and one or more emotional conditions, but not between emotional conditions. The seventh measure—SD of walking right leg swing duration—demonstrated a moderating role of gender. Here, however, there was no significant main effect; rather, gender played a role such that a significant effect was observed only in female participants. More specifically, female participants demonstrated increased SD under the happy condition. To further examine the effectiveness of emotion recognition based on a combination of motion parameters, we applied machine learning models.

### 3.3. Machine learning models

The accuracy, precision, recall, and F1 scores for each of the examined machine learning models are presented in Table 4. The RBF-SVM and the decision tree shared the highest accuracy rate of 45.83%. Because the decision tree had a higher level of interpretability than the RBF-SVM, we explored the features in the decision tree, which utilized 25 of the 229 features with a depth of 10 and 27 leaf nodes to classify the five emotions. The relative importance of each of these features is depicted in Table 5, which shows Gini index values that indicate the priority of a specific class after splitting along a particular measure [86].

**Table 4:** Models and their performance metrics.

Model	Accuracy	Precision	Recall	F1-Score
kNN	0.125	0.11	0.12	0.12
Decision tree	0.458	0.44	0.46	0.44
Logistic regression	0.416	0.37	0.42	0.39
RBF-SVM	0.458	0.42	0.46	0.43
Linear-SVM	0.375	0.30	0.38	0.32
Polynomial-SVM	0.375	0.50	0.38	0.40

**Table 5:** Feature importance (Gini index) based on a decision tree. The term ’cluster’ refers to data after the removal of transition movement.

Motion parameter	Gini index
Walking standard deviation right wrist distance from hip	0.098715
Standing cluster relative standard deviation left wrist distance from hip	0.071407
Walking normalized left mean double-support distance by stance duration	0.066343
Center of pressure relative standard deviation mediolateral (Y) axis based on clustered data	0.066327
Walking normalized right mean step duration	0.056965
Gait initiation normalized swing speed	0.056324
Standard deviation step width based on distance between ankles	0.055795
Walking normalized left mean step speed	0.052463
Walking normalized left mean stance duration	0.045462
Walking left mean stance duration	0.044857
Walking transition standard deviation left wrist distance	0.038818
Center of pressure full velocity standard deviation anterior–posterior (X) axis	0.036747
Walking mean transition shoulder XY angle	0.036281
First step swing width	0.033395
Walking standard deviation back angle	0.032653
Walking standard deviation left wrist distance from hip	0.031746
Gait initiation swing width	0.027706
Walking left standard deviation step duration	0.027211
Standing center of pressure velocity mean mediolateral (Y) axis	0.024187
Standing center of pressure full mean XY axis ratio	0.021769
Standard deviation of step width based on standing center of pressure cluster data	0.020408
Walking normalized right standard deviation swing duration	0.013605
Standing mean step width based on distance between ankles	0.013605
Walking left mean swing duration	0.013605
Walking normalized left mean swing speed	0.013605

**Fig 4:**
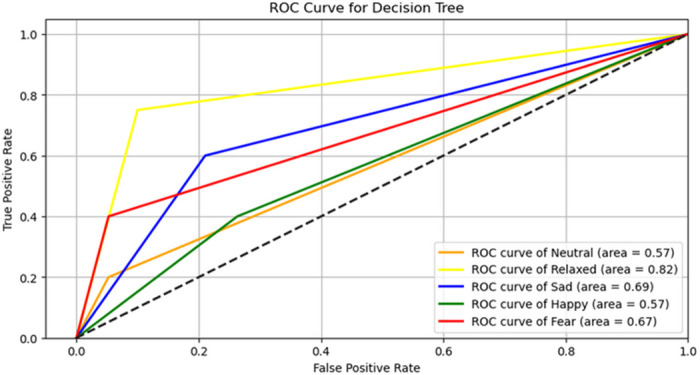
ROC curve for the decision tree. The dotted line shows the performance of the random model. Any point on the graph above the dotted line is considered better than the random model.

The receiver operating curve (ROC) for the decision tree classifier is provided in Fig 4, which shows how true and false positive rates vary for different emotions and suggests that the best prediction would be for the relaxed emotional state. The model predicted relaxed, sad, and fear emotions 32%, 19% and 17% better than random chance, respectively. Fear and relaxed emotions showed the highest precision rates, at 60% or above, while happy showed the lowest rate, of only 29%. Recall for the relaxed emotion was highest, at 75%, followed by sad at 60%, while neutral showed the lowest recall rate, at 20% (Table 6).

**Table 6:** Decision tree emotion classification report.

	Precision	Recall
Neutral	0.50	0.20
Relaxed	0.60	0.75
Sad	0.43	0.60
Happy	0.29	0.40
Fear	0.67	0.40
**Macro Average**	**0.50**	**0.47**
**Weighted Average**	**0.49**	**0.46**

## 4. Discussion

Aiming to improve the accuracy of emotion recognition based on body motion in natural settings, we explored the effects of emotional state on a wide variety of body motion parameters. To this end, we conducted an experiment where participants’ emotional state was manipulated using validated manipulations into five conditions (happiness, relaxation, fear, sadness, and emotionally-neutral). Motion capture system and force plate recorded body posture and movement during *authentic* standing and walking. The data was analyzed using two methods: traditional statistics examining motion parameters separately, and machine learning analyzing a collection of parameters simultaneously.

### 4.1. Effect of emotion on specific motion parameters

Statistical analyses revealed that only 7 of the 229 motion measures demonstrated significant effects for the examined emotions; of the 7 parameters that showed significant effects, 6 showed significant main effects of emotion on motion, and an additional parameter exhibited a moderating role for gender. Interestingly, all the six measures that showed significant effects between neutral and emotional conditions may be interpreted as manifestations of postural control during standing. This is in line with substantial research on the effect of emotion on postural control (e.g., [23, 87]). Yet, the significant differences we observed were mainly between the neutral condition and the relaxed or sadness conditions; these effects are distinct from those obtained in past studies, which have generally suggested that postural response is more salient under states with high emotional arousal [20, 27]. It is important to note that many of the past studies on postural control have examined emotional states related to one’s bodily positions, namely those elicited from factors linked to stability, such as fear of falling, which is considered a *specific* emotion. Such conditions may lead to conscious control of one’s posture [22]. By contrast, the emotional manipulation used in our study evokes general emotional state (often termed mood), which is distinct from *specific* emotions in strength and specificity [88]. The general emotional states in our study do not elicit an urge to control one’s posture, accounting for the discrepancy between our results and those of past studies. Indeed, the distinct results in the current study are in line with Lelard and colleagues’ [23] premise that emotional states elicited by different sources may lead to different effects. Further, the absence of a moderating role for gender in the effect of emotion on factors indicative of postural control is consistent with Horslen and Carpenter’s [20] findings. Our finding that female participants demonstrated increased SD of swing duration of the right leg under happy condition seems to counter research such as [54], in which women were found to be more sensitive to negative events. Yet, it should be acknowledged that only one parameter among 229 exhibited such pattern, and thus it cannot be generalized to gender differences in emotional response.

Importantly, despite the few significant results that point to some consistencies with past studies, the general observation is that many of the significant effects reported in past studies [17, 37, 43, 45, 48, 52] were not observed in the current study. We believe that this fundamental discrepancy may be attributed to two central aspects that distinguish between our study and past research: First, in most past studies the emotions were acted out, which led participants to move in exaggerated manner or in a way reflecting naïve beliefs regarding how emotions should influence body motion. By contrast, in our study the manipulation was more authentic, thus not leading to strong effects. Moreover, the natural setting and the fact that we did not draw participants’ attention to either their emotional state, or their motion, or the link between the two, uncover the real (and not perceptive) motion under the various states. Second, the emotional state in past studies were often determined using observers, which provides indication of the perception of a third party rather than actual feeling, and also limits applicability in reality. Our results of only a few significant effects of emotions on specific motion parameters reinforce findings by Gross [19], who also uncovered only a few significant effects on quantitative measurements of body motion. These consistent observations suggest that single motion parameters may not serve as adequate indicators of emotional state and thus cannot assist in accurate emotion recognition, which echoes a similar premise made by de Meijer [37]. We therefore conclude that the identification of authentic emotions may not be based on single measures but rather on a collection of motion parameters.

### 4.2. Machine learning models for recognizing emotions based on motion

As opposed to traditional statistics considering specific motion parameters separately, our machine learning analysis, which relies on a collection of motion measures, reveals that it is likely to produce models that can yield accurate emotion recognition. Specifically, our results revealed that it is possible to determine five emotional states based on 25 measures with an accuracy of 45.8%, which is more than twice random chance for the five conditions; the decision tree is more interpretative regarding the importance of features. Although past studies of emotion recognition using machine learning have produced higher accuracy rates of 50–80% [2, 3, 42, 43, 45, 52], they relied on acted emotions, which may have been exaggerated, and/or used observers to determine the emotional state, which biases the ground truth in the model assessment. These past studies are, therefore, not applicable in natural settings. Only one study among those that studied emotion recognition using machine learning, as mentioned in our literature review (see Table 1), relied on authentic emotions and determined participants’ state using self-report: Zhao et al. [46], which achieved an average accuracy of 51–74% in predicting depression and anxiety based on participants’ responses on known self-report measures. In addition to the distinct operationalization of emotion, Zhao et al.’s study is different than ours in that it assesses prediction of the level of affective states. That is, emotions in their study are continuous (i.e., levels of depression and anxiety) rather than discrete as in our study. Such an approach is more sensitive and should be considered in the future with a larger variety of affective states.

The results of the machine learning analysis in our study suggested that most features contributing to the model were related to walking posture and COP. It is noteworthy that most past studies that used machine learning for classification did not report the relative importance of each of the features used for the classification (because they used black box methods), and thus, it is impossible to examine whether this observation is consistent with the previous studies.

Finally, comparison between emotional states in the model recognition indicates highest precision for fear and relaxed emotions and lowest for happiness. Past studies revealed inconsistent patterns of recognition rates for the various emotions (e.g., in some studies, recognition of happiness was lowest [50], while in others it was highest [42]). Thus, it is impossible to generalize from our study and past research regarding which emotions are easier to detect.

Altogether, the results of analysis indicated that it is possible to use machine learning methods to obtain emotion classification with when emotions are naturally experienced and expressed. The current research lays the foundation for further development of emotion recognition models and technology. Our experimental setup can be used in studying the effect of natural emotions on motion, using more sophisticated models such as neural networks, which may exhibit better performance.

### 4.3. Limitations and future directions

It should be acknowledged that our results rely on a relatively small sample size, which, according to sensitivity analysis, can detect medium or large effects on single parameters. Further studies with larger sample sizes may detect smaller effects if they exist. In addition, our machine learning model assessed non-person-specific emotion recognition. Yet, previous research suggested that person-specific models incorporating body motion or gait parameters are more accurate (95%) for classifying emotions as compared to non-person-specific models (69%) [2]. It may very well be, therefore, that integrating person-specific emotion recognition with the approach employed in our study will achieve higher accuracy rates. Future research could examine this option, recognizing the contexts in which person-specific models might be applicable. Further consideration of various machine learning models also merits examination. Future research could also examine the effects of emotions on a wider variety of body motions simultaneously, and possibly extend the examination to include other movements that were not included in the current research, such as segment rotation; this would require a more complex marker model. This line of research should also be extended to different manipulations that might produce stronger emotional reactions than movie segments, as well as to additional emotions.

## 5. Conclusion

This research suggests that relying on single specific motion parameters cannot assist in emotion recognition. Instead, machine learning models that consider multiple motion parameters are a more viable approach. More research should be conducted to refine the recognition model and to enhance accuracy. Looking forward, once the motion variables needed for classification have been identified, they could serve in a guideline for future use with less accurate measurements. Recent technological developments (e.g., Microsoft Kinect and Intel Real Sense depth cameras [89, 90] or pose estimation tools using regular RGB cameras to detect the human skeleton in 2D and 3D [91]), albeit less accurate, may be applicable for emotion recognition in the home setting. Finally, an examination of similar effects among the elderly population also merits attention due to the potential application of such findings to the development of elder-assisting devices.

## Supporting information

S1 Data (ZIP)

S1 Appendix Motion parameters used in analyses. (DOCX)

S2 Appendix Results of single parameter analysis. (DOCX)
