# Supplementary material for: Emotion and motion: Toward emotion recognition based on standing and walking
Source: PLoS One. 2023 Sep 13;18(9):e0290564. doi: 10.1371/journal.pone.0290564 (PMC10499259; doi:10.1371/journal.pone.0290564)
Supplement: S1 Appendix — (DOCX) [file pone.0290564.s002.docx]

**Appendix 1. Motion parameters used in analyses**

| **Movement** | **Description** | **Parameters (calculated both as cluster and full data)** | **Balance** | **Standing posture** | **Gait initialization** | **Walking** |
| --- | --- | --- | --- | --- | --- | --- |
| Center of pressure | Point where the total sum of the pressure field acts on a body projected onto the ground | Mean of X axis (anterior and posterior), mean of Y axis (mediolateral), mean of XY axis, mean of XY ratio, standard deviation of X axis, standard deviation of Y axis, standard deviation of XY axis, standard deviation of XY ratio, mean velocity of X axis, mean velocity of Y axis, mean velocity of XY axis, mean velocity of XY ratio, standard deviation velocity of X axis, standard deviation velocity of Y axis, standard deviation velocity of XY axis, standard deviation velocity of XY ratio, number of transitions | x |  |  |  |
| Step width | Distance between the ankle markers in two consecutive steps | Mean, standard deviation |  |  |  | x |
| Base of support | Area beneath a person that includes every point of contact between them and the ground; the area enclosed by heel, ankle, and toe markers | Mean, standard deviation | x |  |  |  |
| Body angles | Angles between different body markers on different axes | Mean of shoulder angle on XY (transverse) axis, mean of shoulder angle on XZ (sagittal) axis, mean of head angle, mean of back angle, standard deviation of shoulder angle in XY axis, standard deviation of shoulder angle in XZ axis, standard deviation of head angle, standard deviation of back angle | x | x |  | x |
|  |  |  |  |  |  | Contd. |
| **Movement** | **Description** | **Parameters (calculated both as cluster and full data)** | **Balance** | **Standing posture** | **Gait initialization** | **Walking** |
| Swing height | Maximum height reached by the ankle marker during the swing phase of a gait cycle relative to the floor | Mean, standard deviation |  |  | x | x |
| Swing distance | Distance between the toe-off and heel strike of the same leg during a swing cycle | Mean, standard deviation |  |  | x | x |
| Swing duration | Duration of the swing phase during a gait cycle | Mean, standard deviation |  |  | x | x |
| Swing speed | Speed achieved during the swing phase of a gait cycle | Mean, standard deviation |  |  | x | x |
| Double- support distance | Distance between the ankle markers of both legs at consequent toe-off | Mean, standard deviation |  |  |  | x |
| Stance duration | Duration of the stance phase during a gait cycle | Mean, standard deviation |  |  |  | x |
| Double-support distance by stance duration | Double-support distance divided by stance duration | Mean, standard deviation |  |  |  | x |
| Temporal ratio | Swing duration divided by stance duration | Mean, standard deviation |  |  |  | x |
| Step length | Distance between consecutive heel strikes | Mean, standard deviation |  |  |  | x |
| Step duration | Duration of one gait cycle | Mean, standard deviation |  |  |  | x |
| Distance | Distance between wrist and hip | Mean, standard deviation |  | x |  | x |
