# Supplementary material for: Emotion and motion: Toward emotion recognition based on standing and walking
Source: PLoS One. 2023 Sep 13;18(9):e0290564. doi: 10.1371/journal.pone.0290564 (PMC10499259; doi:10.1371/journal.pone.0290564)
Supplement: S2 Appendix — (DOCX) [file pone.0290564.s003.docx]

**Appendix 2. Results of single parameter analysis**

| Motion parameter | Effect of emotional state on motion parameter | Role of gender in the effect of emotional state on motion parameter (⯀male ⯀female) | Description of findings |
| --- | --- | --- | --- |
| COP (SD in mediolateral plane) during standing | 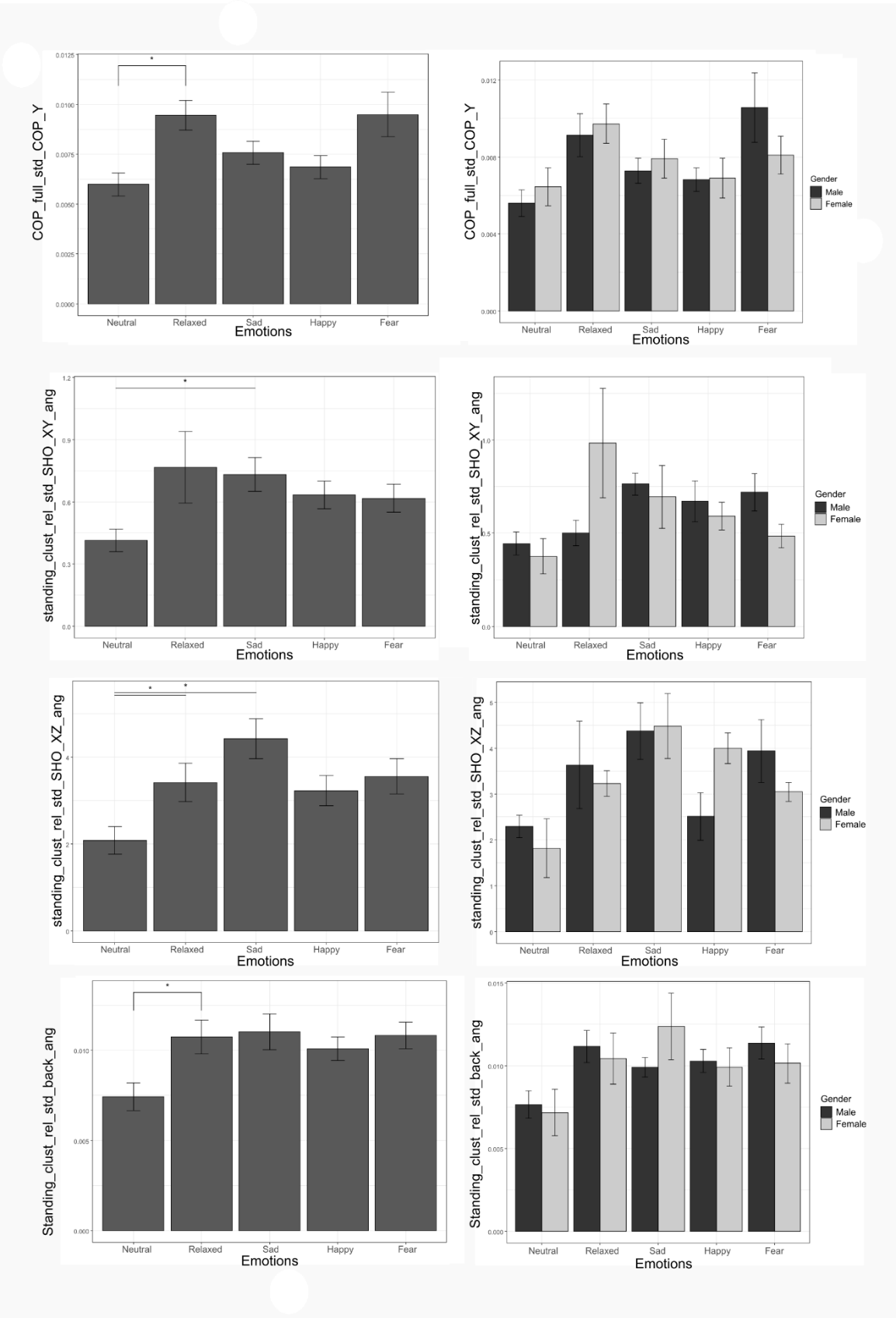 | 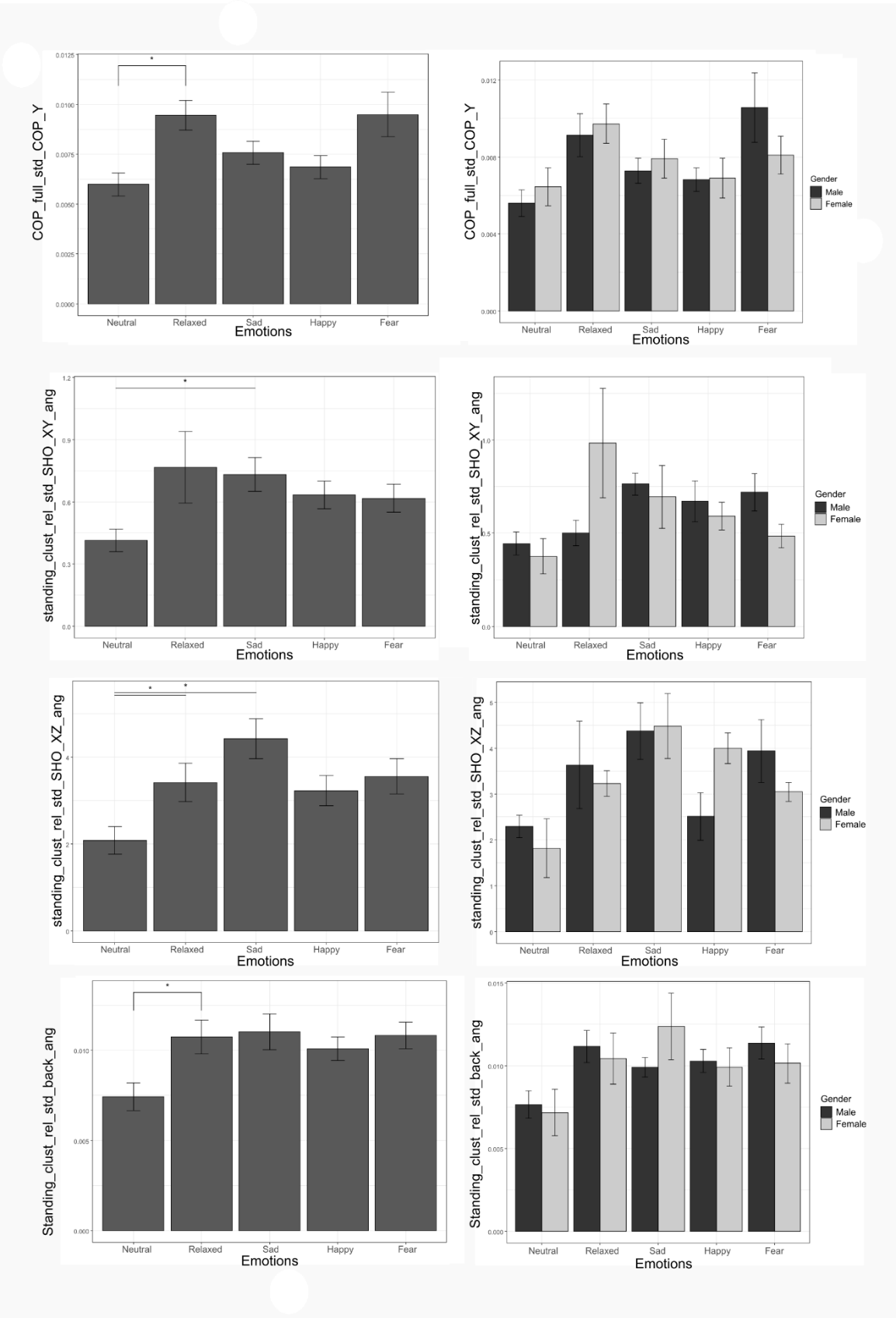 | The SD of the COP while standing revealed significant differences between emotional conditions (*p* = 0.01). Pairwise comparisons showed a significant difference between the neutral and relaxed conditions (*M*_neutral_ = 0.005, *M*_relax_ = 0.009; *p* = 0.04). COP variability in the mediolateral increased by 55% for the relaxed state as compared to neutral. |
| Standing shoulder XY angle (SD in sagittal plane) | 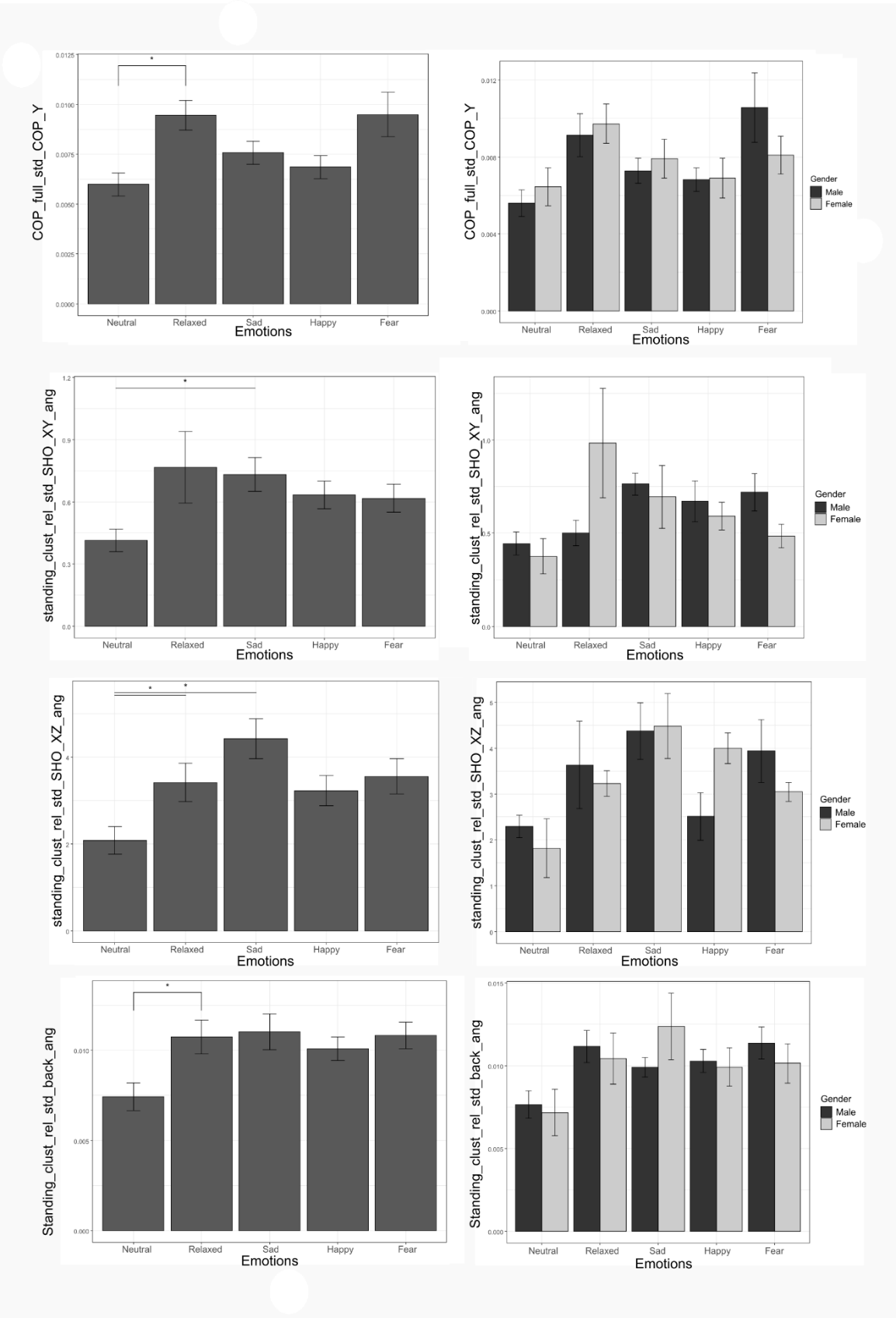 | 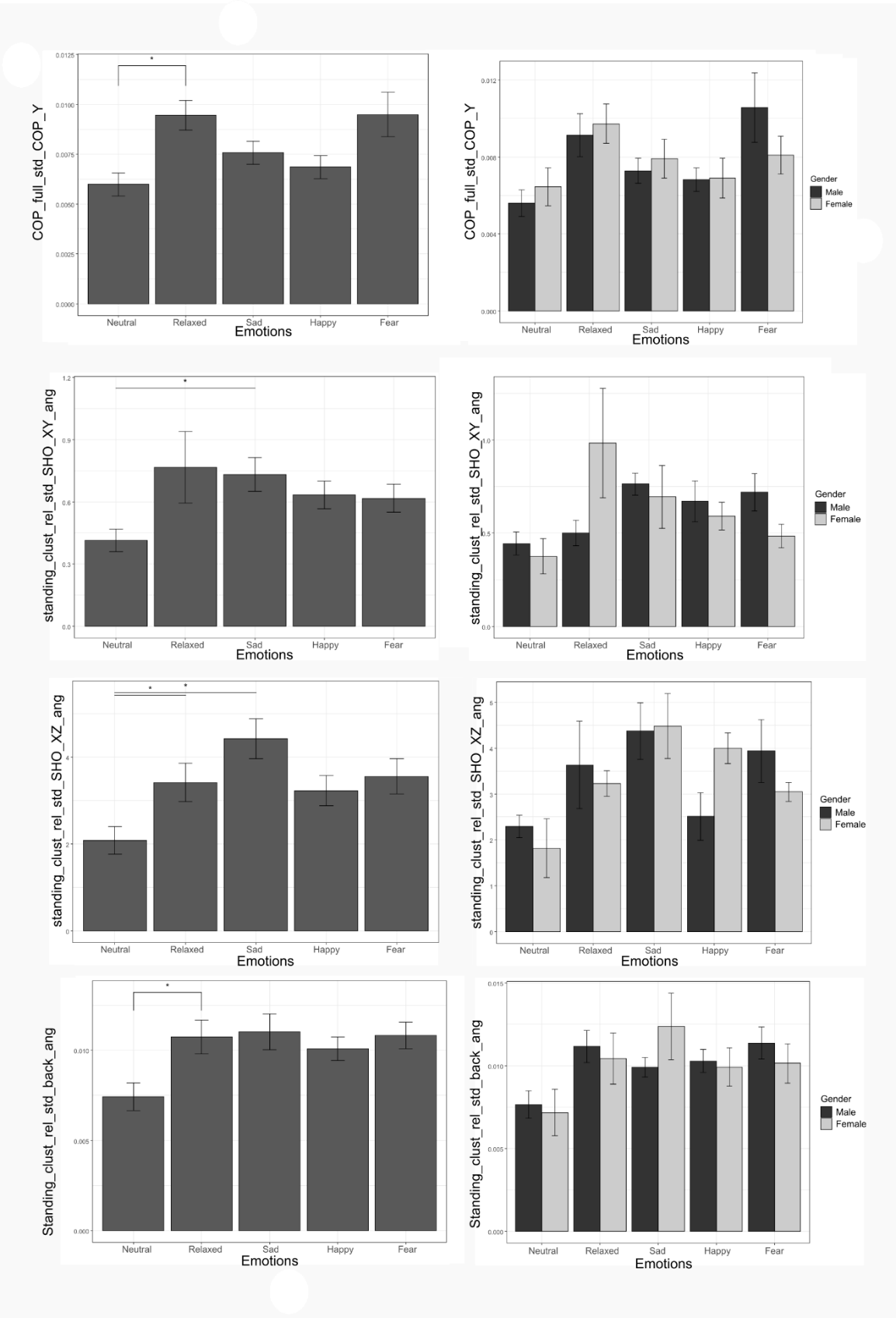 | The SD of the shoulder XY angle while standing revealed marginally significant differences between emotional conditions (*p* = 0.06). Pairwise comparisons revealed a significant difference between the neutral and sad conditions (*M*_neutral_ = 0.40, *M*_sad_ = 0.71; *p* = 0.05), indicating a 79% increase in variability of shoulder movement in the anterior–posterior position during the sad state as compared to neutral. |

| Motion parameter | Effect of emotional state on motion parameter | Role of gender in the effect of emotional state on motion parameter (⯀male ⯀female) | Description of findings |
| --- | --- | --- | --- |
| Standing shoulder XZ angle (SD in frontal plane) | 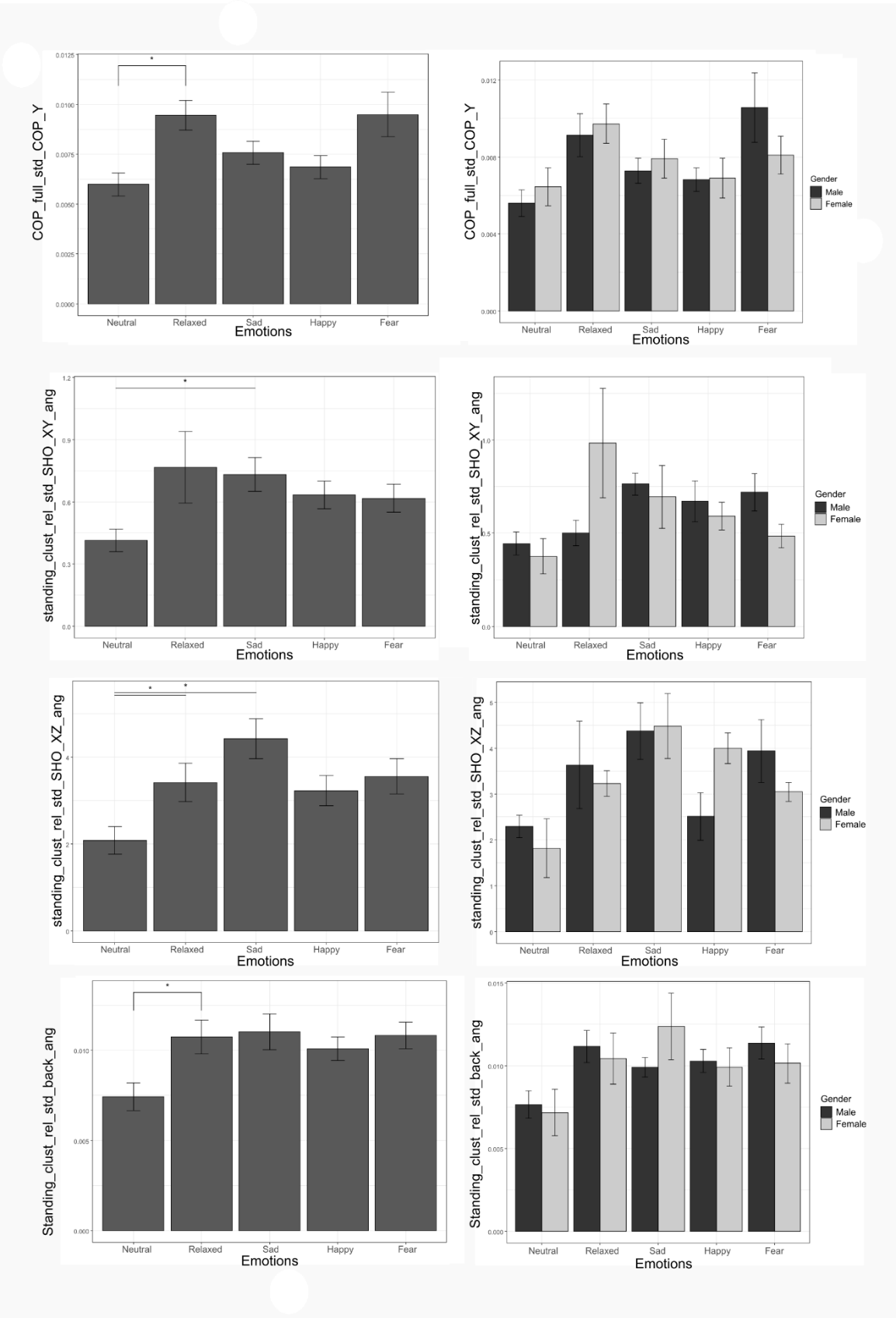 | 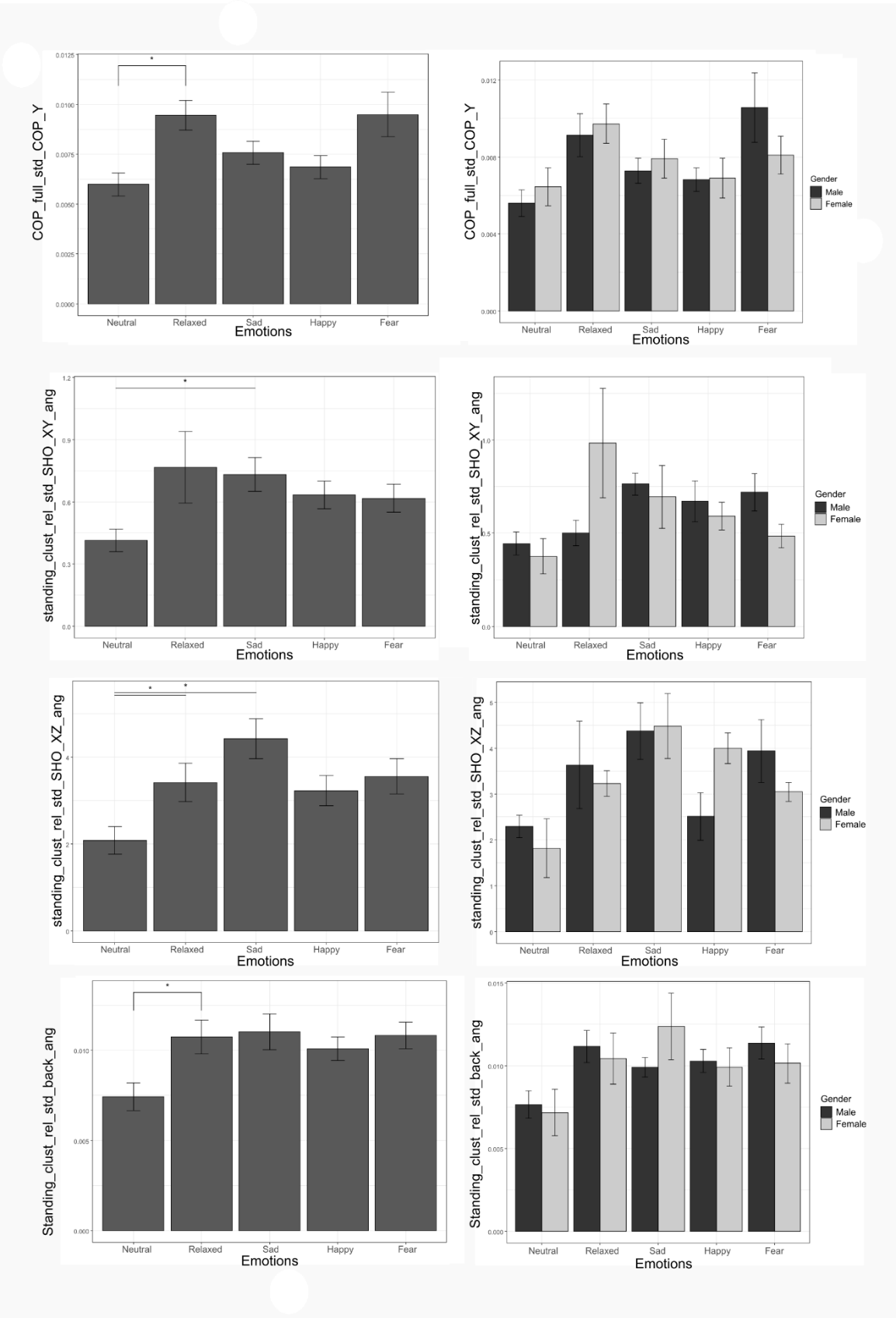 | The SD of the shoulder XZ angle while standing revealed significant differences between emotional conditions (*p* = 0.01). Pairwise comparisons showed a significant difference between the neutral and relaxed conditions (*M*_neutral_ = 2.03, *M*_relax_ = 3.78; *p* = 0.04) and between the neutral and sad conditions (*M*_neutral_ = 2.03, *M*_sad_ = 4.37; *p* = 0.04). As compared to the neutral state, there was a 115% increase in shoulder movement variability during the sad state and an 86% increase during the relaxed state. |
| Standing back angle (SD in sagittal plane) | 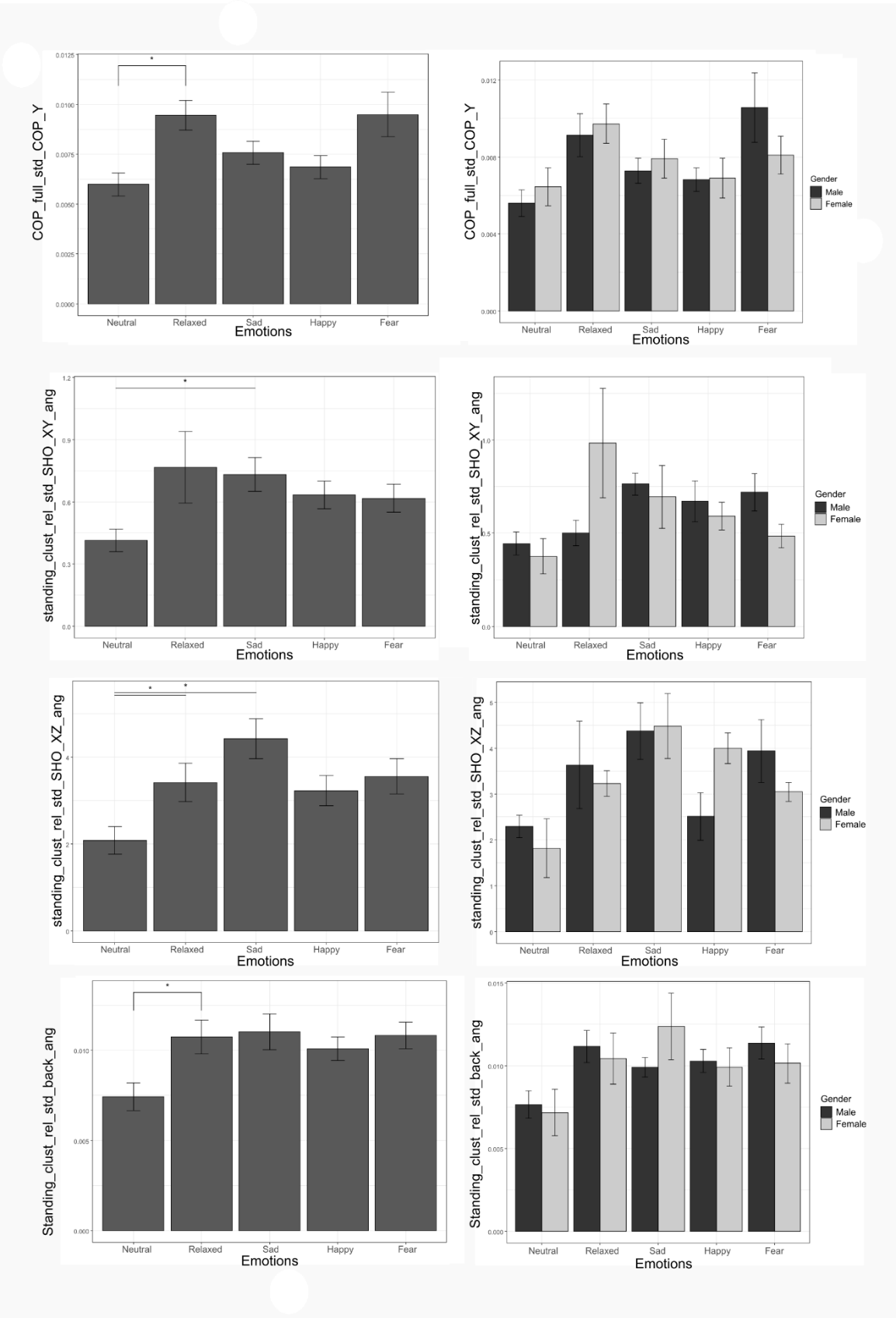 | 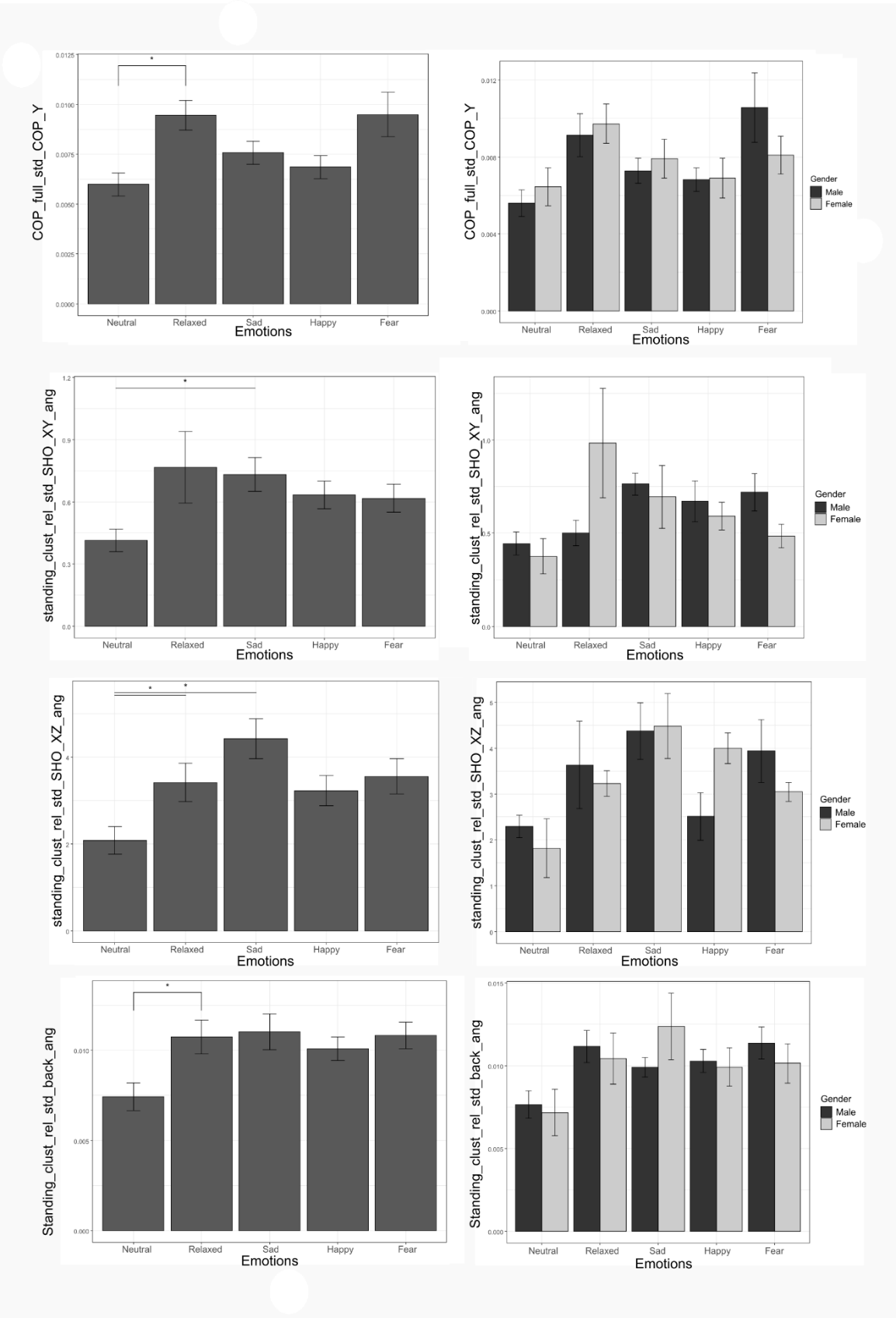 | The SD of the back angle while standing revealed significant differences between emotional conditions (*p* = 0.02). Pairwise comparisons showed a significant difference between the neutral and relaxed conditions (*M*_neutral_ = 0.07, *M*_relax_ = 0.11; *p =*0.04), indicating a 52% increase in back angle variability during the relaxed state as compared to neutral. |

| Motion parameter | Effect of emotional state on motion parameter | Role of gender in the effect of emotional state on motion parameter (⯀male ⯀female) | Description of findings |
| --- | --- | --- | --- |
| SD of standing left wrist to hip distance | **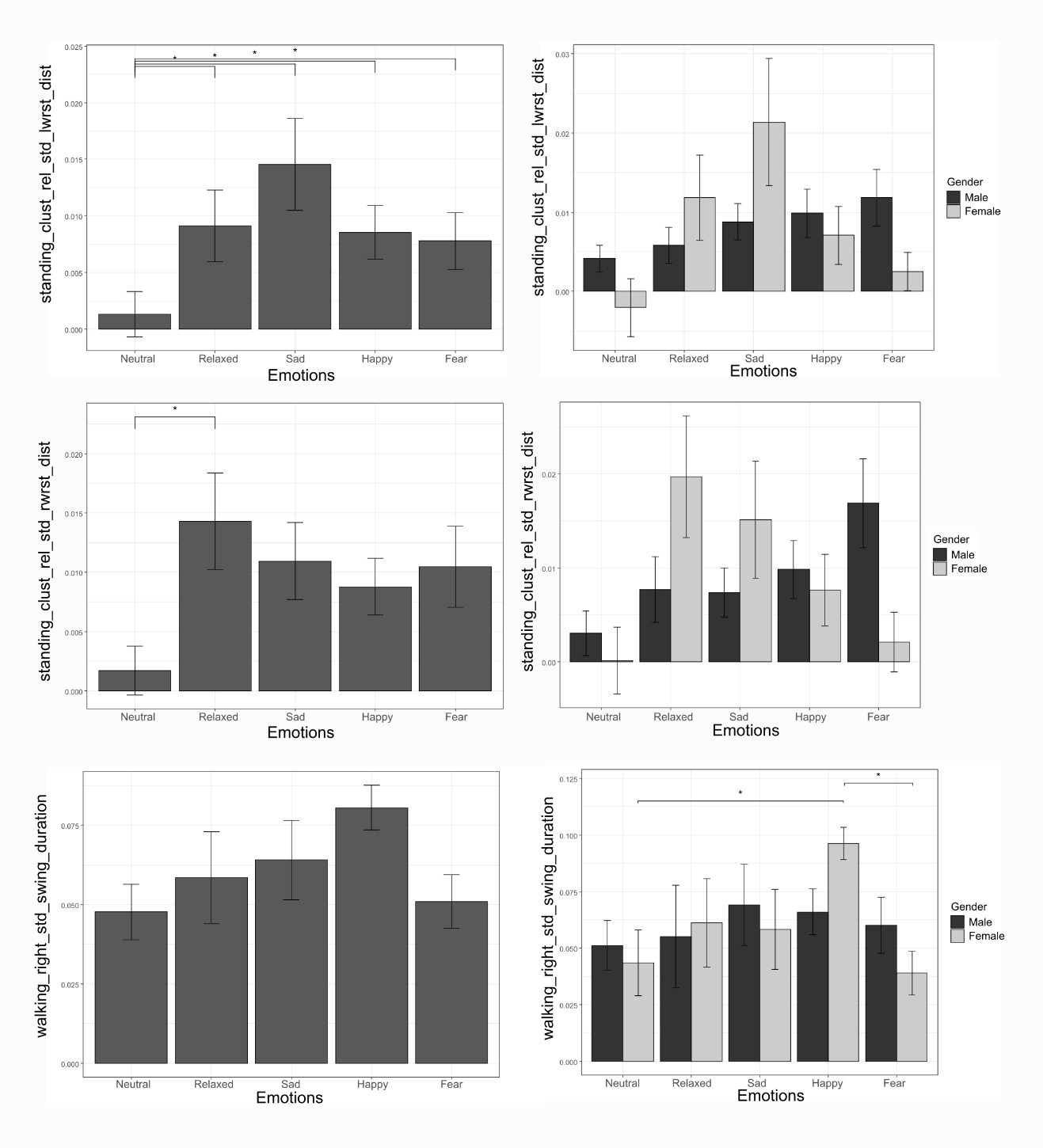** | **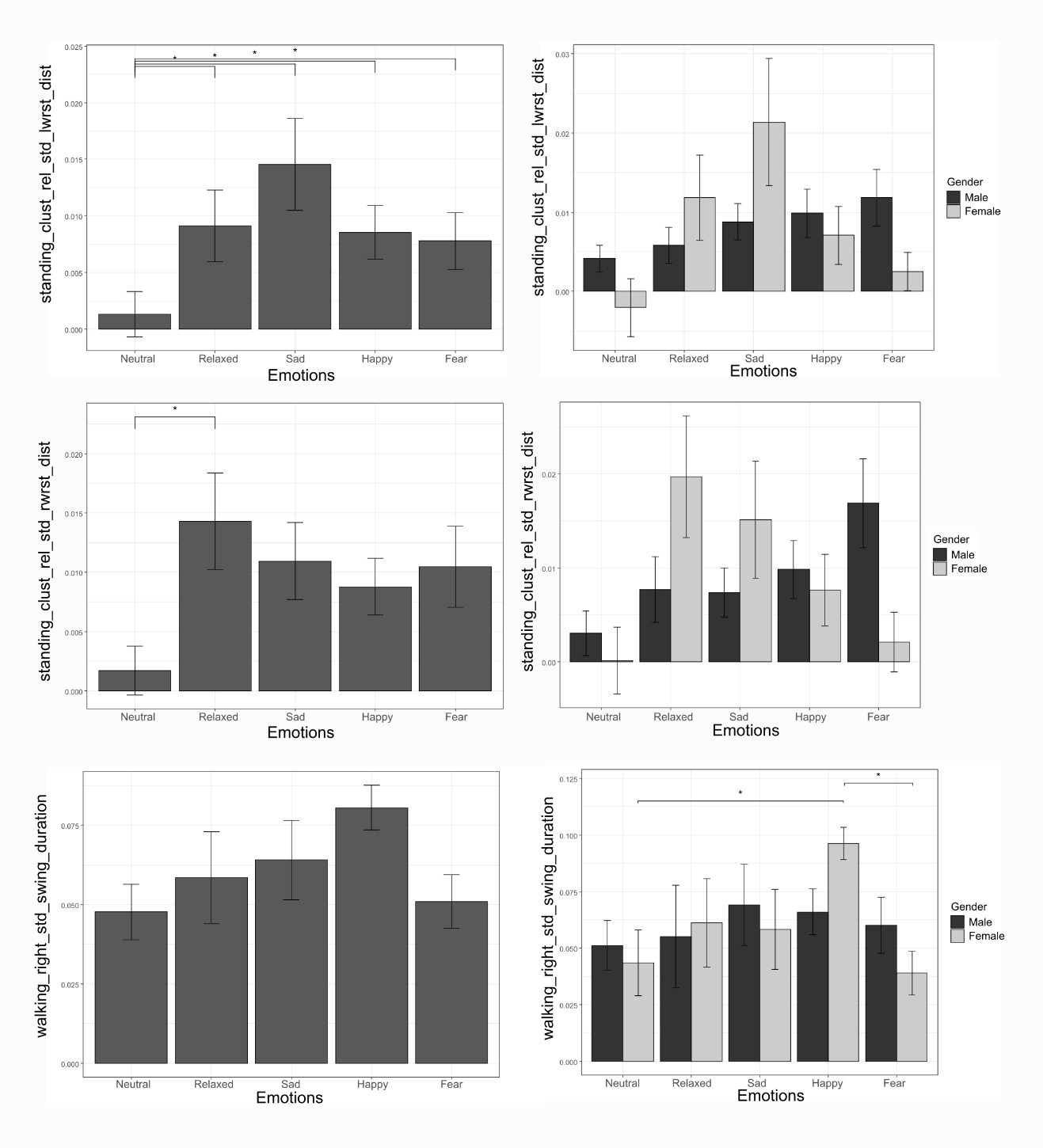** | The results for the SD of this distance indicated a significant difference between emotional conditions (*p* = 0.03). Pairwise comparisons showed significant differences between the neutral and relaxed conditions (*M*_neutral_ = 0.001, *M*_relax_ = 0.009; *p* = 0.03), the neutral and sad conditions (*M*_neutral_ = 0.001, *M*_sad_ = 0.014; *p* = 0.03), the neutral and happy conditions (*M*_neutral_ = 0.001, *M*_happy_ = 0.008; *p* = 0.03), and the neutral and fear conditions (*M*_neutral_ = 0.001, *M*_fear_ = 0.007; *p* = 0.03). These results showed an average increase of 700% in the variability of the distance from the left wrist to the hip during all emotional states as compared to neutral. |
| SD of standing right wrist to hip distance | **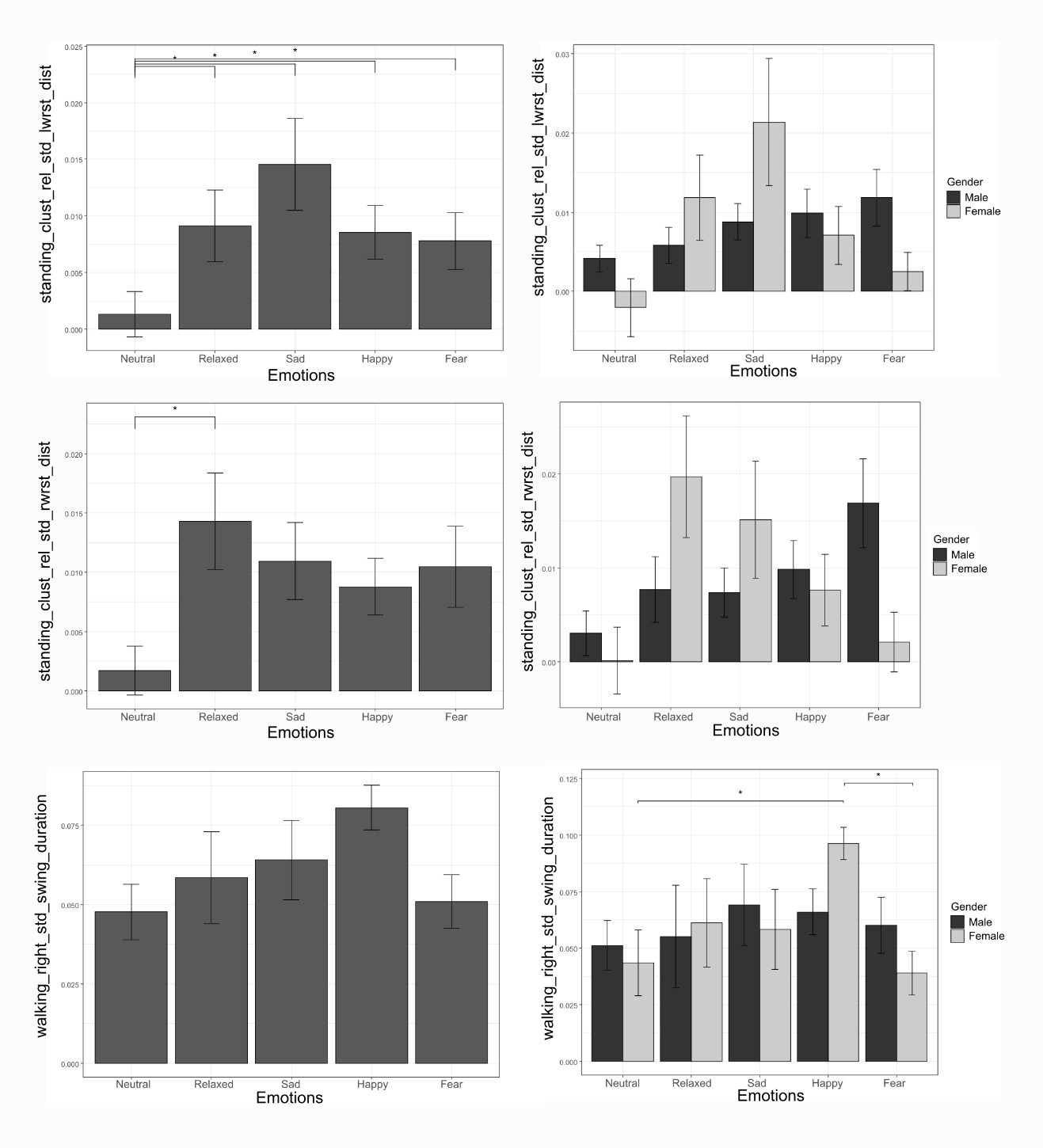** | **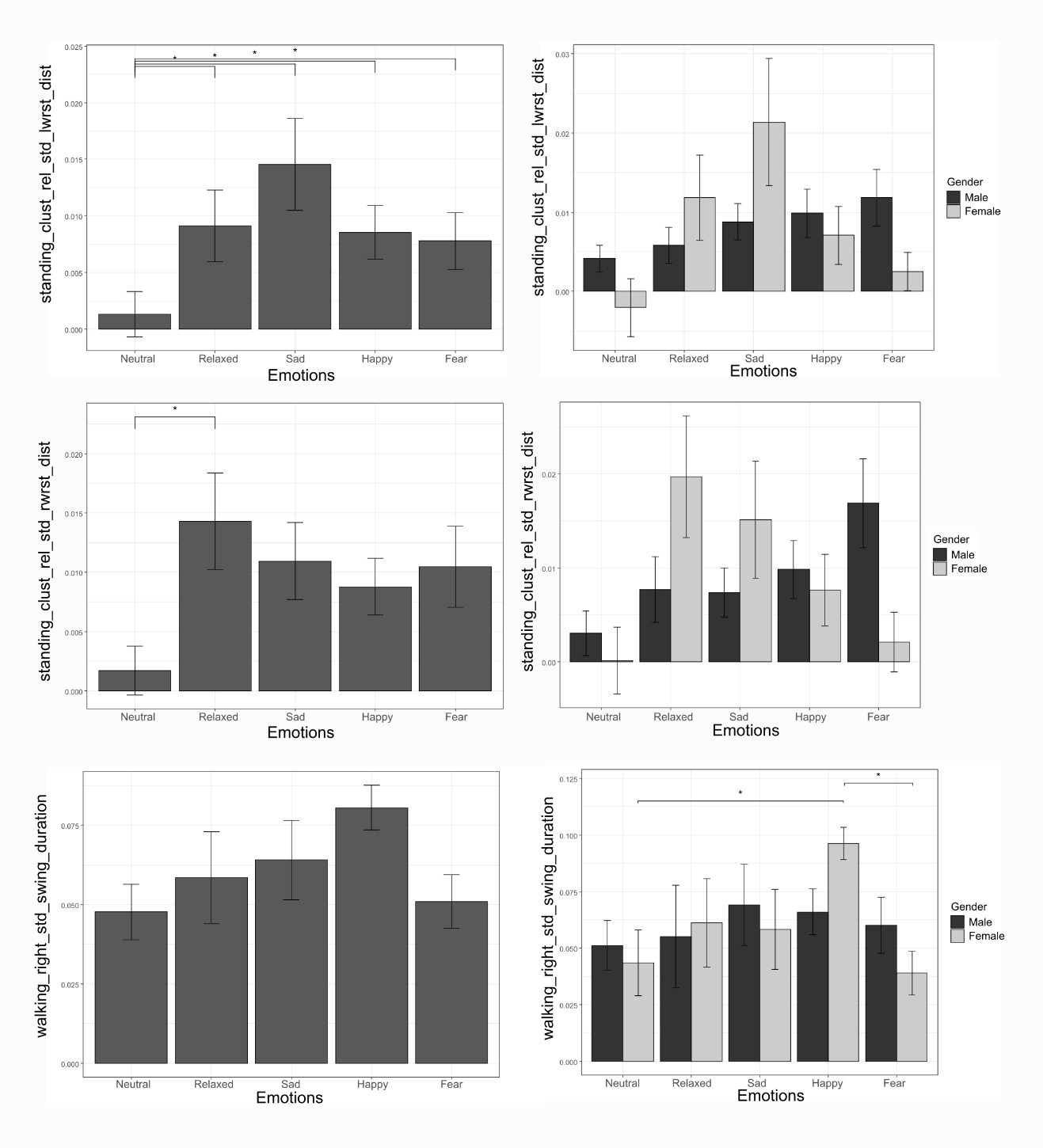** | The effect of emotions on the SD of the right wrist distance while standing revealed significant differences between emotional conditions (*p* = 0.01). Pairwise comparisons showed a significant difference between the neutral and relaxed conditions (*M*_neutral_ = 0.001, *M*_relax_ = 0.014; *p* = 0.03) and an average increase of 896% in the variability of the distance from the right wrist to the hip during the relaxed emotional state as compared to neutral. |

| Motion parameter | Effect of emotional state on motion parameter | Role of gender in the effect of emotional state on motion parameter (⯀male ⯀female) | Description of findings |
| --- | --- | --- | --- |
| SD of walking right leg swing duration | **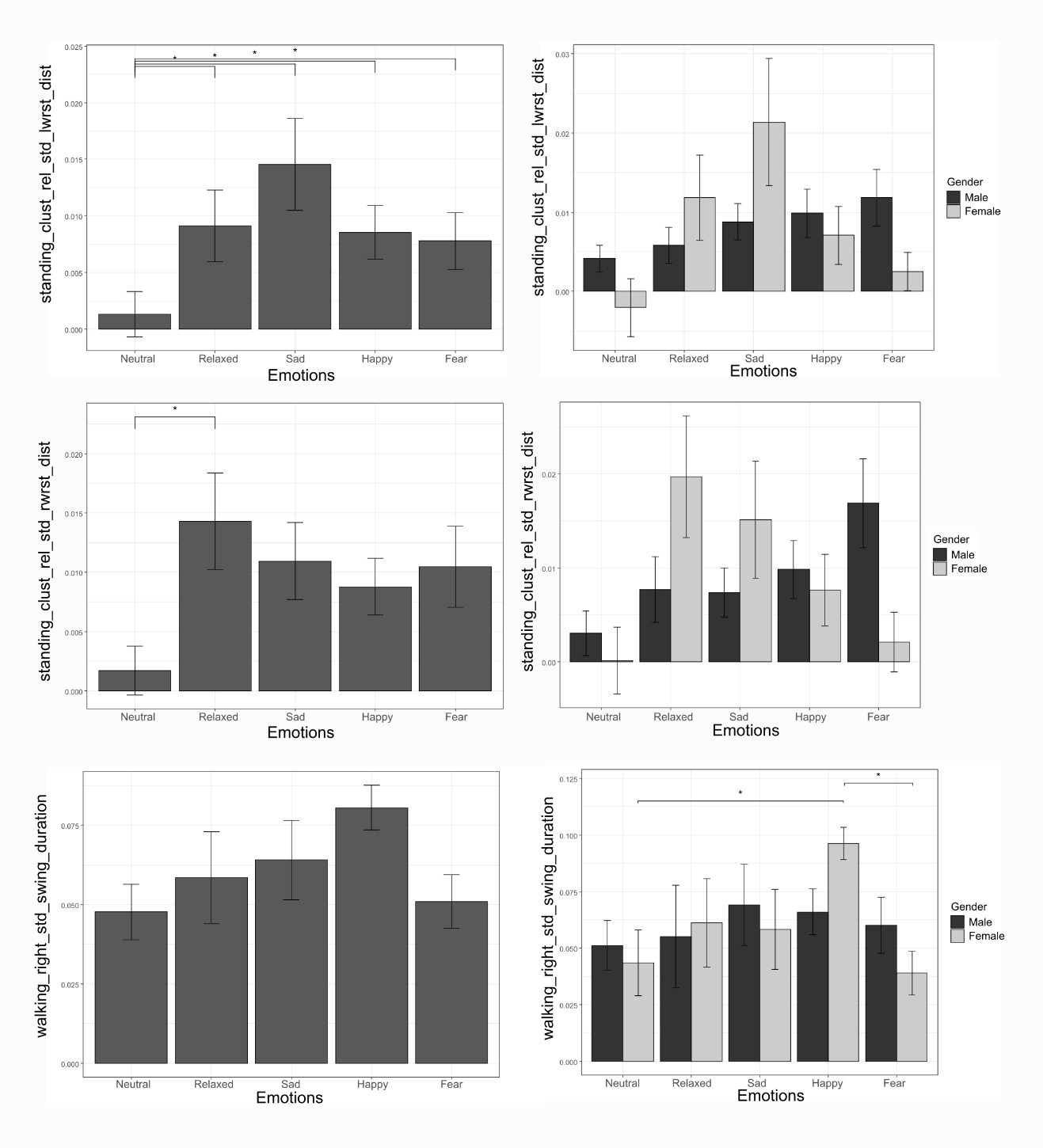** | **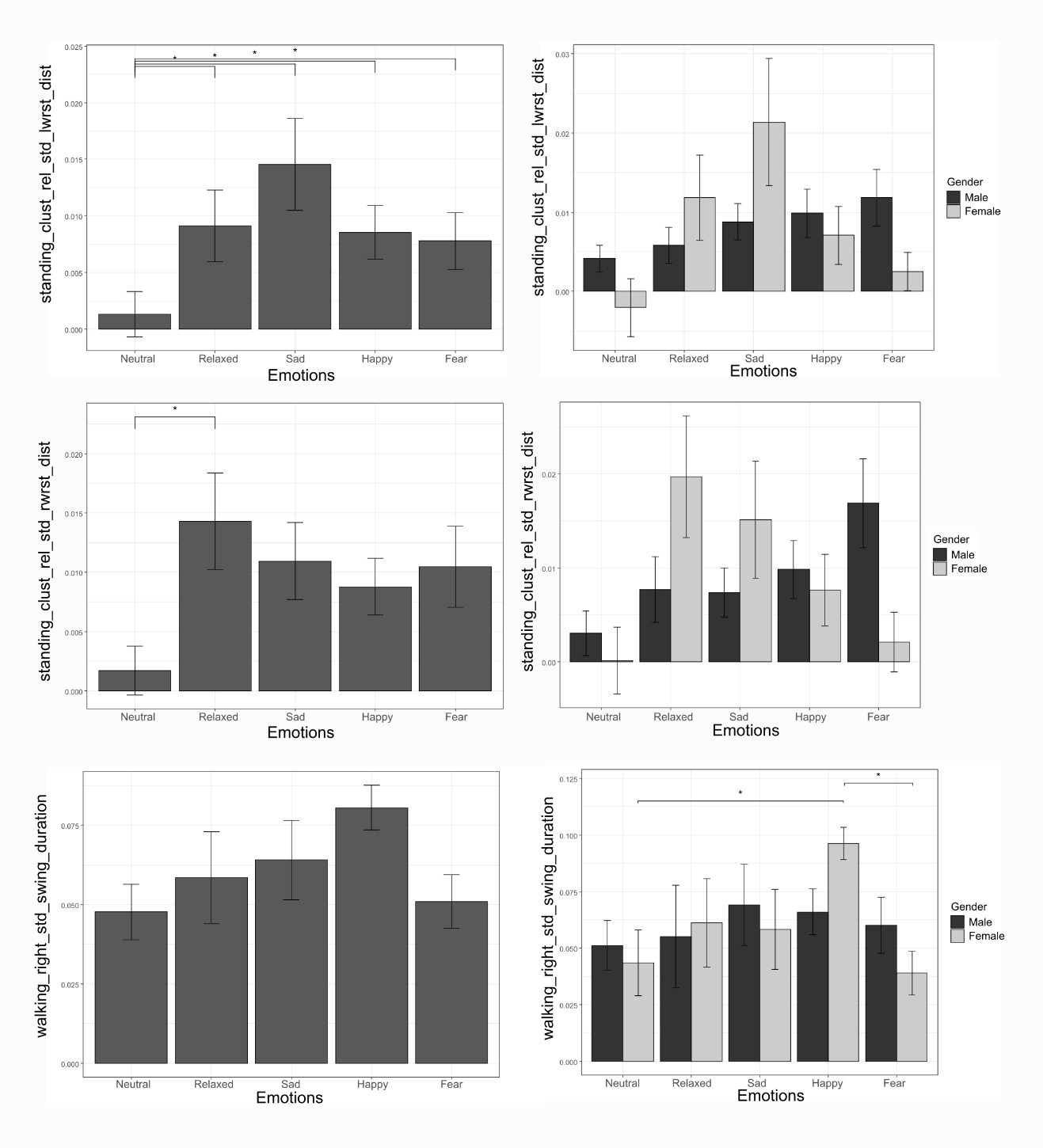** | The SD of swing duration while standing revealed no significant differences between emotional conditions (*p* = 0.23). Two-way ANOVA, however, showed that swing duration was affected by emotions differently across genders (*p* = 0.02). Pairwise comparisons showed significant differences between the neutral and happy conditions (*M*_neutral_ = 0.04, *M*_happy_ = 0.09; *p* = 0.04) and the fear and happy conditions (*M*_fear_ = 0.03, *M*_happy_ = 0.09; *p* = 0.04) for female participants, while male participants did not show any significant differences in swing duration across conditions. |
